# COVID-19 Test Allocation Strategy to Mitigate SARS-CoV-2 Infections across School Districts

**DOI:** 10.3201/eid2903.220761

**Published:** 2023-03

**Authors:** Remy Pasco, Kaitlyn Johnson, Spencer J. Fox, Kelly A. Pierce, Maureen Johnson-León, Michael Lachmann, David P. Morton, Lauren Ancel Meyers

**Affiliations:** The University of Texas at Austin, Austin, Texas, USA (R. Pasco, K. Johnson, S.J. Fox, K.A. Pierce, M. Johnson-León, L.A. Meyers);; Santa Fe Institute, Santa Fe, New Mexico, USA (M. Lachmann, L.A. Meyers);; Northwestern University, Evanston, Illinois, USA (D.P. Morton)

**Keywords:** COVID-19, respiratory infections, severe acute respiratory syndrome coronavirus 2, SARS-CoV-2, SARS, coronavirus disease, zoonoses, viruses, coronavirus, epidemiological modeling, asymptomatic screening, optimization, public health response, United States

## Abstract

In response to COVID-19, schools across the United States closed in early 2020; many did not fully reopen until late 2021. Although regular testing of asymptomatic students, teachers, and staff can reduce transmission risks, few school systems consistently used proactive testing to safeguard return to classrooms. Socioeconomically diverse public school districts might vary testing levels across campuses to ensure fair, effective use of limited resources. We describe a test allocation approach to reduce overall infections and disparities across school districts. Using a model of SARS-CoV-2 transmission in schools fit to data from a large metropolitan school district in Texas, we reduced incidence between the highest and lowest risk schools from a 5.6-fold difference under proportional test allocation to 1.8-fold difference under our optimized test allocation. This approach provides a roadmap to help school districts deploy proactive testing and mitigate risks of future SARS-CoV-2 variants and other pathogen threats.

By early January 2023, the COVID-19 pandemic had caused >6.7 million deaths worldwide (*1*), and severe socioeconomic hardship (*2*–*4*), particularly for racial minorities (*5*,*6*). Children experienced pandemic-related school closures, that led to substantial losses in learning (*7*–*9*), elevated rates of child abuse (*10*), lack of access to healthy food (*11*), and emotional harm (*12*). By the end of March 2020, all kindergarten through 12th grade (K–12) public schools in the United States had stopped in-person instruction (*13*), affecting 55 million students. Schools in 48 US states remained closed through the end of the school year (*14*). In August and September of 2020, a total of 74% of the 100 largest school districts in the United States started the year with remote-only teaching (*15*). By November 2020, 19% of those districts remained fully remote, and 36% had fully resumed in-person learning (*15*). Schools continued to reopen throughout the year.

By September 2021, a large fraction (70%) of US adults had been vaccinated with highly effective SARS-CoV-2 vaccines (*16*), including an estimated 86% of K–12 teachers and school staff (*17*). However, children <12 years of age were still ineligible for vaccines (*16*,*18*). Because large pockets of the country were still unvaccinated, COVID-19 continued to claim lives, and 100,000 deaths were reported in the United States during July–September 2021, including 246 deaths among children 0–17 years of age (*16*). At the end of October 2021, the United States authorized administration of COVID-19 vaccines for children 5–11 years of age (*19*). In January 2022, schools returned from winter break amidst a major COVID-19 wave fueled by the emergence of the highly transmissible and immune-evasive Omicron variant (F.P. Lyngse, unpub. data, https://doi.org/10.1101/2021.12.27.21268278), and case counts among students and staff reached record numbers despite increasing vaccine coverage in the United States (*16*,*20*).

Schools across the country adopted diverse reopening plans for the 2021–22 school year. Among the largest districts, 96% offered some form of in-person learning (*21*). Although some schools fully returned to prepandemic normal operations without COVID-19 interventions, many adopted policies for using, social distancing, quarantine, or testing requirements to safeguard the return to campus. The federal government invested $122 billion to support safe, in-person instruction through screening, improved building ventilation, purchase of personal protective equipment, hiring of additional personnel, and other measures (*22*,*23*). Within the first 2 months of the school year, ≈1.5% of schools closed temporarily in response to COVID-19 outbreaks (*21*).

Frequent and systematic testing of asymptomatic persons has been shown to be a viable and cost-effective mitigation strategy in communities, universities, and schools (*24*–*29*). However, tests are costly and inaccessible for many school districts in the United States; districts with limited testing resources must determine how to allocate testing across schools to protect their students, staff, and communities. Some districts have opted to restrict testing to symptomatic persons and other districts have apportioned tests according to school enrollment (*30*,*31*).

In this study, we propose a strategy for allocating testing resources across a diverse school district in which the frequency of testing depends on the school’s enrollment and grade range, recent COVID-19 cases reported among students and staff, and the estimated prevalence in the surrounding (i.e., catchment) community. Coupling derivative-free constrained optimization and a detailed agent-based simulation of SARS-CoV-2 transmission within and between classrooms, we derived an optimal allocation of tests across a school system that could minimize the maximum risk for cumulative infections on any campus over a 10-week period. We applied our approach to design a testing strategy for the 11 main high schools in the Austin Independent School District (AISD; Austin, Texas, USA), which has 18,500 enrolled students and 1,500 staff (*32*).

## Methods

To determine an optimal allocation of tests across schools we developed a 2-step framework in which we first modeled COVID-19 transmission within schools for different levels of surveillance testing and then used those results as an input to an optimization model (Appendix). We first considered a hypothetical school system with 6 schools of 500 students each that differ over 2 parameters: community incidence and in-school transmission rate. For community incidence, we considered low and high scenarios. In the low scenario, the community had 35 new daily cases/100,000 persons; in the high scenario the community had 70 new daily cases/100,000 persons. For the transmission rate, we considered unmitigated R_0_ values to be low (1.0), moderate (1.5), or high (2.0).

We then modeled 11 high schools in AISD by using student enrollment based on attendance in early January 2021. We considered 2 different in-school transmission rate scenarios: an unmitigated transmission rate in all schools of with an R_0_ of 1.0; and transmission rates of each school estimated by fitting a regression model of the number of cases reported in that school against the estimated enrollment (*33*) (Appendix).

For each school and each scenario, we ran 300 stochastic simulations. We assumed that only students (not adult staff) were tested on Monday mornings and that test results were available instantly (*34*); preliminary analysis suggested that testing adults had minimal effects. During any given week, students in the model were selected for testing evenly across classes rather than testing a subset of entire classes.

In addition to proactive testing, we assumed that 90% of symptomatic persons would seek testing 0.5–1.5 days after symptom onset and then isolate after testing, even before results are available. We assumed 20% of infected students and 57% of infected adults would become symptomatic (*35*,*36*).

In our analysis of a hypothetical school system, we assumed that tests were perfectly accurate and that a positive test immediately triggered a 14-day isolation of the person and a 14-day quarantine of their household and classroom members. For our case study of AISD, we assumed lower test accuracy based on estimates for the widely used Abbott BinaxNow antigen tests (https://www.abbott.com), which had 95% sensitivity for symptomatic persons, 80% sensitivity for asymptomatic persons, and 99% specificity (*37*,*38*).

### Transmission Model

We built a stochastic agent-based model of COVID-19 transmission within schools that included household transmission for students. We held the average community incidence constant through the simulation and all persons could become infected through outside interactions that were not explicitly modeled. The modeled population included students, teachers, staff, bus drivers, and members of the students’ households. We modeled various contacts between agents in schools (Appendix).

We used published estimates for the average SARS-CoV-2 incubation, latent, and infectious periods, as well as a person’s infectiousness through time (*39*). We assumed that asymptomatic cases were two thirds as infectious as symptomatic cases (*40*).

We simulated contacts at half-hour intervals and stochastically determined infection events based on the transmission probability between pairs of interacting persons. For each scenario, we derived the transmission rate to produce the specified unmitigated in-school R_0_ (Appendix). This R_0_ is the basic reproductive rate we would obtain without any testing, symptomatic or surveillance, and reflects other mitigation measures in place, such as face masks or social distancing.

We ran simulations for 10 weeks. We initiated simulations with everyone susceptible and simulated community and household infections for 10 days before school started.

### Optimization Model

The objective of our test-allocation problem was to minimize the maximum risk experienced by any school in the system under consideration. Because of the stochastic nature of our COVID-19 transmission model, we had to choose a measure that summarized the risk for a given school under each possible frequency of surveillance testing. We defined the risk for each school as the expected number of on-campus infections of students plus the 90% conditional value-at-risk (CVaR) of the number of on-campus infections. Here, CVaR represents the expected number of such infections, conditional on restricting attention to the worst 10% of simulated outcomes, and hence accounts for risk aversion. Given 2 candidate allocations with a similar average number of cases, we preferred the allocation that limited the upside tail risk in terms of a large outbreak. We further accounted for risk by taking the worst-case risk measure across all schools. We used on-campus infections, rather than total infections, because total infections are partially driven by community incidence rather than school interventions. We further used the proportion of a school’s infected population rather than the absolute number of infections, which enabled us to treat large and small schools equally. Then, we could calculate each school’s risk as a function of the number of tests allocated; more tests reduced the risk incurred. Our goal was to allocate tests to schools to minimize the largest risk measure incurred at any school, subject to the constraints that we respect total testing capacity across the school system and that we cannot test all students at a school more than once per week (Appendix Figure 13).

## Results

**Figure 1:**
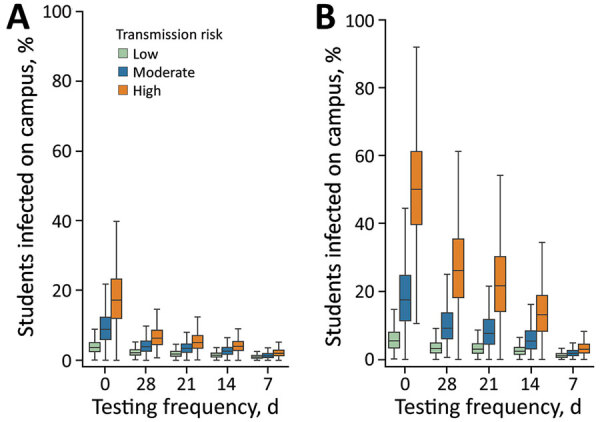
Projected effects of a COVID-19 test allocation strategy to mitigate SARS-CoV-2 infections across 11 school districts in the Austin Independent School District, Austin, Texas, USA. The whisker plots demonstrate projected effects over a 10-week period in a school with 500 students under 2 scenarios: A) assuming the household and classroom of each detected case is quarantined; or B) assuming only households (not entire classrooms) are quarantined. Colors indicate reproduction numbers as low (1.0), moderate (1.5), and high (2.0) in-school transmission risks in the absence of proactive or symptomatic testing, isolation, and quarantine. Whiskers indicate points that lie within 1.5 interquartile ranges of the lower and upper quartiles; boxes indicate interquartile range and horizontal bars indicate median fraction of students infected on-campus depending on the frequency of proactive testing as never (0), or once per every 28, 21, 14, or 7 days. Results are based on 300 stochastic simulations for each scenario.

Under all transmission scenarios, we expected proactive testing to greatly reduce the proportion of students infected on campus over a 10-week period (Figure 1, panel A). In the high-risk scenario (in-school R_0_ = 2.0), 14-day testing reduced the fraction of students infected from 18.2% to 4.1%; under the lowest risk scenario (in-school R_0_ = 1.0), the expected incidence decreased from 4.0% to 1.5%. When we increased testing frequency from every 14 days to every 7 days, the expected incidence in high-risk scenario reduced to 2.1% and expected incidence in low-risk scenario reduced to 1.0%.

**Figure 2:**
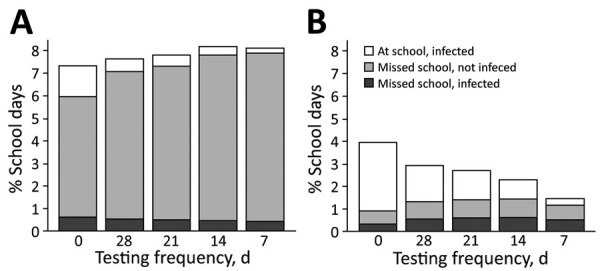
Projected days of school missed in a COVID-19 test allocation strategy to mitigate SARS-CoV-2 infections across 11 school districts in the Austin Independent School District, Austin, Texas, USA. The graphs demonstrate the expected proportion of school days missed due to isolation or quarantine over a 10-week period in a school with 500 students under 2 scenarios: A) assuming the household and classroom of each detected case is quarantined; or B) assuming only households (not entire classrooms) are quarantined. Estimates assume a moderate (reproduction number = 1.5) in-school transmission risk in the absence of proactive or symptomatic testing, isolation, and quarantine. All projections assume that isolation and quarantine periods last 14 days. In addition to on-campus transmission, persons might be exposed in the surrounding community at a rate of 35 new daily infections/100,000 population. The results are based on 300 stochastic simulations for each scenario.

The efficacy of testing to mitigate in-school transmission depended on whether quarantine was limited to the household of the positive case or extended to the entire classroom (Figure 1, panel B). Under a moderate transmission scenario (in-school R_0_ = 1.5) in which students are tested every other week, the expected incidence decreased from 6.3% (95% CI 1.0%–15.6%) to 2.9% (95% CI 1.0%–6.2%) when we added classroom quarantine to household quarantine. We also estimated the costs of quarantine in terms of days of in-school education lost over the 10-week projection period, under the moderate transmission risk scenario (Figure 2, panel A; Appendix Figure 11). Without proactive testing, we expected the strategy of quarantining entire classrooms after a positive test to result in an average of 3 (6%) out of 50 school days missed per student. We expected household-only quarantine to reduce that cost by roughly 6.5-fold, to an average 0.9% of school days missed; however, that strategy roughly doubled the days students spent at school while infectious (Figure 2, panel B). 

Regardless of quarantine policy, our model showed that proactive testing could reduce in-school exposure, with few to no additional lost days of school. In addition, we found that shortening the quarantine period for classroom contacts from 14 to 7 days would mitigate some of the educational losses without substantially increasing health risks (Appendix Figure 12).

As a sensitivity analysis, we also considered a higher rate of SARS-CoV-2 introductions from the surrounding community by raising daily new cases from 35 cases/100,000 persons to 70 cases/100,000 persons and lowering the accuracy of SARS-CoV-2 tests (*37*,*38*) (Appendix Figures 10, 11). In our sensitivity analysis, we found that our estimates were robust to the assumed sensitivity and specificity of the tests (Appendix).

### Case Study—Optimizing Testing across a Large Municipal School District

**Figure 3:**
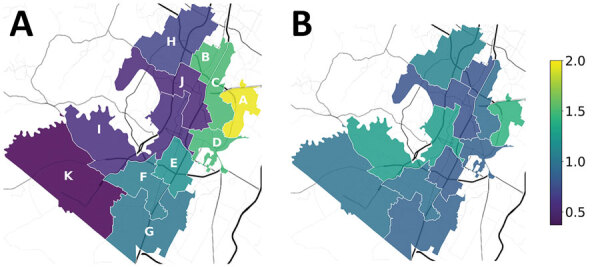
Locations of 11 high schools in the Austin Independent School District, Austin, Texas, USA, used to model a COVID-19 test allocation strategy to mitigate SARS-CoV-2 infections across school districts. A) Daily incidence of COVID-19 infections in late January 2021 in the catchment area of each high school relative to the average incidence across the Austin Metropolitan Statistical Area. Estimates are based on COVID-19 case reports during March 2020–January 2021. A value of one corresponds to the average incidence in the MSA. Schools are listed A through K from highest to lowest estimated daily incidence (Appendix Table 3). B) On-campus transmission risks, estimated from reported COVID-19 cases during August 16, 2020–March 8, 2021. Values are scaled so that 1.0 means that the school reported the expected number of cases, based on a least-squares linear fit of reported cases to school enrollment (Appendix Figure 4).

We applied our model to derive an optimal allocation of testing resources across the 11 high schools in AISD, the largest district in Austin, Texas, which includes 75,000 students, 5,500 teachers, and 5,000 staff. The district operates 125 schools from pre-K–12th grade; 55% of students are Hispanic and 30% White, and >50% come from economically disadvantaged backgrounds (*32*). We estimated the external force of infection for each school by comparing reported COVID-19 incidence in the neighborhood of a school to reported incidence across the entire metropolitan statistical area (MSA) from March 2020–January 2021 (Figure 3, panel A) (*41*). We listed the schools in order of the estimated external risks; the catchment of school A had almost double (195%) the city-wide incidence, and the catchment of school K had only 37%. In general, risk (i.e., COVID-19 incidence) was higher on the east side of Austin.

We estimated on-campus transmission risk for each school by using reported cases from each school during August 16, 2020–March 8, 2021 (Figure 3, panel B). In brief, we scaled the in-school R_0_ based on the difference between the cumulative, per student incidence in a school to the cumulative incidence throughout the district. We assumed a baseline R_0_ of 1.0; thus, schools with incidence equal to the district-wide incidence had resulting estimates that ranged from 0.70–1.41. Our estimates for on-campus transmission risk and external force of infection were not greatly correlated (Appendix). We also ran scenarios in which all schools had the same transmission risk (Appendix Figures 5, 6).

**Figure 4:**
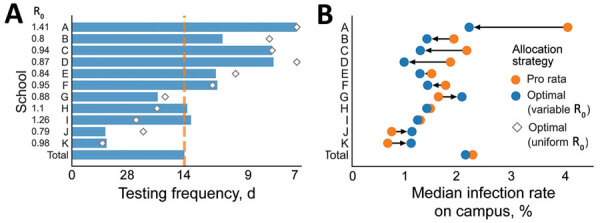
Test allocations and estimated infection rates based on testing frequency in a COVID-19 test allocation strategy to mitigate SARS-CoV-2 infections across 11 school districts in the Austin Independent School District, Austin, Texas, USA. A) Testing allocation for 3 testing strategies. Orange dashed line indicates pro rata strategy; blue bars indicate optimized strategy to minimize the maximum risk; diamonds indicate optimized strategy considering only variation in community transmission risks. Numbers to the left of the y-axis indicate the assumed on-campus reproduction number for each school. B) The median percent of students infected on-campus under the optimized strategy (blue) and pro rata strategy (orange), over a 10-week period; arrows indicate increases or decreases in infection rates. We modeled infections rates by using 3 testing strategies: pro rata, in which all schools test their students once per every 14 days; optimized to minimize the maximum risk of any school, considering variation in both community and in-school transmission risks; optimized considering only variation in community transmission risks. Values are averaged across 300 simulations (Appendix Table 4). The model assumes that classrooms quarantine for 14 days following a positive test.

On the basis of the estimated heterogeneity in risks across the district, we estimated the optimal allocation of testing resources across schools by searching the space of possible allocations. For a given allocation, we projected the outcome for each school by first averaging the expected cumulative incidence (i.e., the mean across 300 simulations) and then the projected tail risk (i.e., the mean across the 10% worst-outcome simulations). We found the maximum value across schools (i.e., the projection for the highest-risk school) and then selected the allocation that minimized this value. Assuming that the average community incidence was 70 new daily cases per 100,000 population, based on estimates from late January 2021 in the Austin area (*42*), and that the district had a total testing budget of 1 test per student every 14 days across the district, the optimized allocation ranged from testing once per 45 days in the lowest-risk school (school K) to once per 7 days in the highest-risk school (school A) (Figure 4, panel A). We assumed that testing could not be administered more frequently than weekly. The optimal allocation differed slightly when we assumed instead that schools had the same on-campus R_0_ and differences in risk stemmed solely from the community force of infection (Figure 4, panel A).

We projected infection rates under both the optimized allocation and a nonoptimized pro rata allocation in which resources would be allocated proportional to enrollment (Figure 4, panel A). We expected the optimized strategy to slightly reduce the overall infection rate for the district relative to the pro rata strategy and equalize risks across campuses. In the optimized strategy, the median infection rate increased by 0.4% for the lowest-risk school (school K) and decreased by 1.8% for the highest-risk school (school A) (Appendix Table 4).

**Figure 5:**
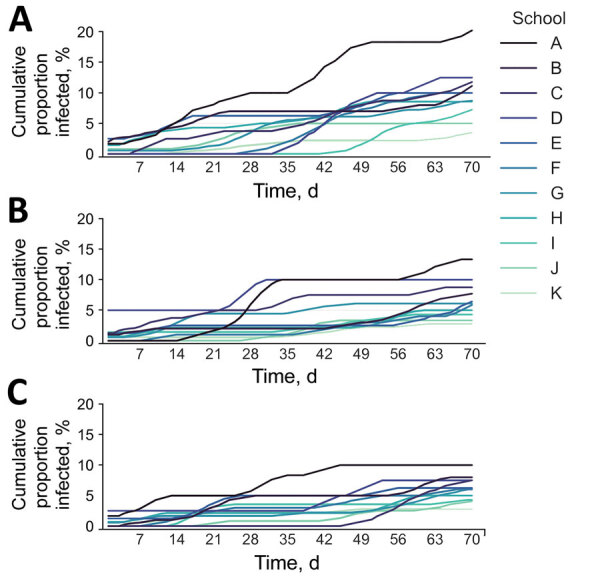
Cumulative infections in schools used to model a COVID-19 test allocation strategy to mitigate SARS-CoV-2 infections across 11 school districts in the Austin Independent School District, Austin, Texas, USA. Graphs represent cumulative COVID-19 infections over a 10-week period under 3 testing scenarios: A) no testing; B) all schools test students every 14 days; and C) optimized allocation of tests based on school-specific transmission risks, assuming a district-wide budget of 1 test per student every 14 days. Schools are ordered from A–K based on community incidence from high to low in the school catchment area. Graphs show 7-day moving averages based on a single simulation for each scenario and school. To show representative projections, we selected the simulation that produced a cumulative attack rate closest to the median across all 300 simulations.

When we considered total incidence by combining both community-acquired and school-acquired infections, we expected ≈5.8-fold difference between the highest risk and lowest risk schools, in the absence of testing (Figure 5, panel A). Using a 14-day testing budget, we found a pro rata strategy would lower overall incidence but not reduce the disparity (Figure 5, panel B), but an optimized allocation would greatly shrink the gap to a 3.6-fold difference (Figure 5, panel C). Restricting our analysis to infections that occur on campus, the optimized allocation again reduced the disparity in risk across schools (Table).

**Table:** Estimated heterogeneity in COVID-19 incidence and total disease burden across 11 high schools in the Austin Independent School District, Austin, Texas, USA, under 3 testing scenarios in a modeled COVID-19 test allocation strategy to mitigate SARS-CoV-2 infections across school districts*

Infections	Testing allocation		
	No testing	Pro rata testing	Optimal testing
Total infections†			
Risk gap	5.8	4.8	3.6
Gini coefficient (SE)‡	0.23 (0.053)	0.26 (0.057)	0.19 (0.037)
No. infections (95% CI)§	115 (79–158)	70 (50–94)	69 (49–93)
Infection rate (95% CI)§	9.4 (6.5–12.9)	5.7 (4.1–7.7)	5.6 (4–7.6)
On-campus infections#			
Risk gap	6.5	5.6	1.8
Gini coefficient (SE)‡	0.27 (0.098)	0.23 (0.075)	0.13 (0.041)
No. infections (95% CI)§	70 (38–119)	27 (13–49)	26 (12–48)
Infection rate (95% CI)§	5.8 (3.1–9.7)	2.2 (1.1–4.1)	2.1 (1.0–3.9)

*The risk gap is the ratio of the median cumulative incidence across 300 simulations of the school with the highest expected incidence to that of the school with the lowest expected incidence. †Total student infections, occurring both on and off campus, over the 10-week projection period. ‡Gini coefficients indicate overall disparities in expected burden, where values of 0 correspond to maximum equality and values of 1 correspond to maximum inequality (*43*). Gini coefficients were calculated using the median proportion of students infected across 300 simulations. §The total median infections in the district over the horizon simulated, expressed as absolute and per capita. #Infections occurring on campus, over the 10-week projection period.

To provide intuition, we also derived an optimal testing allocation to reduce risks in a hypothetical district containing 6 schools, 1 of each combination of either low or high external risk and either low, moderate, or high internal risk (Appendix Figures 8, 9). We compared 3 possible testing scenarios: no testing, universal testing every 2 weeks, and an optimal testing strategy in which the 2-week testing budget is allocated to schools to minimize the maximum risk experienced by any school in the system. We found that going from no testing to a pro rata allocation decreased the maximum risk for any school from 24.7% (95% CI 11.9–38.0) of students infected to 6.6% (95% CI 3.0–11.4); under the optimal allocation, risk was further reduced to 4.5% (95% CI 1.4–8.3). Using this strategy, the total expected risk across all 6 schools was reduced from 12.8% (95% CI 9.0–16.9) of infections without testing to 3.8% (95% CI 2.5–5.2) with a pro rata allocation, which was further reduced to 3.5% (95% CI 2.4–4.8) under the optimal testing allocation (Appendix Figure 14, panel B).

## Discussion

Proactive testing can be an effective strategy for preventing SARS-CoV-2 transmission on school campuses, if test turnaround is short and positive cases are immediately isolated (*44*; A. Bilinski, unpub. data, https://doi.org/10.1101/2021.05.12.21257131). Because testing requires considerable time, resources, and personnel, schools might opt to streamline their efforts as COVID-19 risks change. Our study provides a framework to help school districts allocate limited testing resources across different schools, depending on the in-school and local community transmission risks, while weighing the costs and benefits of classroom quarantine after a positive test. Prioritizing testing based on estimated risks can help mitigate the disproportionate COVID-19 burden falling on lower socioeconomic and racial minority neighborhoods (*45*–*47*).

Our results suggest that the optimal allocation of tests across schools depends on both the in-school transmission rate and the force of infection from the surrounding community. However, estimating in-school risks is difficult without sufficient testing because of overdispersion in the distribution of secondary cases and the small proportion of children that develop symptoms upon SARS-CoV-2 infection (*48*). A modest level of baseline surveillance testing could help determine the relative risks across schools (*49*). Our case study of AISD high schools suggests that even without such information, allocating testing resources based on community risks alone could substantially close gaps among schools (Appendix Figure 7).

Although proactive testing can lower and equalize COVID-19 risks across a heterogeneous school district, disparities are likely to persist. Schools drawing from neighborhoods with high COVID-19 incidence will continue to experience higher case counts and absenteeism. Other intervention measures, including vaccination and use of face masks, are essential for further reducing risks and ensuring equitable access to education.

The optimal allocation of scarce resources across multiple entities, like the number of tests per school, depends on the state of the entire system. A school might receive anywhere from no tests to enough tests for weekly testing of every student, depending on the level of risk relative to other schools. Schools could potentially game the system to gain larger allocations. For example, a school could inflate reported cases or enable higher rates of transmission by allowing high-risk activities or relaxing precautionary measures. If such issues arose, then allocation calculations could be based solely on estimates for the force of infection from the surrounding communities.

This approach can be broadly applied to distributing limited SARS-CoV-2 testing resources across systems with heterogeneous risks, such as workplaces, correctional facilities, or long-term care facilities. Our case study demonstrates that, even within a single city, tailoring control strategies to hyperlocal estimates of risks can reduce transmission overall and mitigate chronic disparities in access to resources and disease burden. On larger geographic scales, spatiotemporal variation in COVID-19 risks has been even more apparent, and cities, states, and countries exhibit highly asynchronous waves of transmission. Dynamic allocation of scarce public health resources based on reliable estimates of risk could substantially reduce the burden of COVID-19 and future pathogen threats across the United States but requires considerable coordination at the state and federal level.

The first limitation of our study is that we assumed immediate in-school and community risks could be reliably estimated. In practice, the data required to estimate such risks often lag, are biased, or are unavailable. Such uncertainty could be included in our model by using stochastic variables that evolve based on test results from each school. However, the additional complexity would slow computational optimization. Second, we estimated heterogeneity in incidence but did not explicitly consider vaccination, health outcomes other than incidence, or socioeconomic or other factors known to correlate with COVID-19 risks (*50*). Schools drawing from more vulnerable communities might have access to fewer mitigation resources besides testing, lower vaccination coverage, or higher infection hospitalization and mortality rates. Such factors could be explicitly modeled and incorporated into the objective function used to derive equitable allocations. Third, the study derived allocations to minimize infections occurring across a school district. However, other outcomes could be explicitly incorporated into further analyses, including absenteeism and loss of education resulting from isolation and quarantine. The costs and benefits of quarantining entire classrooms, in addition to the households of positive cases, depend on the frequency of testing. Classroom quarantine would always be expected to elevate absenteeism but only substantially reduces exposure risks when testing is infrequent. With frequent testing or low transmission risks, limiting the scope and duration of quarantine might be advisable. Hospitalization risks for school staff and the potential for schools to exacerbate transmission in the surrounding community also could be integrated into allocation calculations. Finally, our model does not consider the potential costs or logistical impediments to dynamically allocating tests among schools. In addition to the challenges of rapidly calculating allocations and distributing tests accordingly, schools might require additional trained staff to administer tests, conduct contact tracing, and ensure the quick and safe isolation and quarantine of affected persons (*30*).

In conclusion, as the United States plans for COVID-19 postpandemic management, proactive testing will remain a highly effective countermeasure that can be tailored to changing risks on a local scale. As tests become more economical and as surveillance within schools and communities improves, our model demonstrates that school systems can optimize testing and quarantine policies to prevent transmission, limit absenteeism, and ensure continuity of operations during future COVID-19 surges.

Appendix Additional information on COVID-19 test allocation strategy to mitigate SARS-CoV-2 infections across school districts.
