# Supplementary material for: COVID-19 Test Allocation Strategy to Mitigate SARS-CoV-2 Infections across School Districts
Source: Emerg Infect Dis. 2023 Mar;29(3):501–10. doi: 10.3201/eid2903.220761 (PMC9973671; doi:10.3201/eid2903.220761)
Supplement: Appendix — Additional information on COVID-19 test allocation strategy to mitigate SARS-CoV-2 infections across school districts. [file 22-0761-Techapp-s1.pdf]

Article DOI: https://doi.org/10.3201/eid2903.220761

# COVID-19 Test Allocation Strategy to Mitigate SARS-CoV-2 Infections across School Districts

## Appendix

## 1. Transmission Model

We developed an agent-based model of COVID-19 transmission in schools, where we explicitly model interactions between students and adults working in the school environment during school days. We also include students' households in our simulation while broader community interactions are modeled through random daily introductions of new cases. In our hypothetical examples we consider schools with 500 students spread across 25 classrooms, with 25 teachers, 25 bus drivers, and 16 other school staff. The entire framework, both the transmission and the optimization models, were implemented in Python version 3.8.8 (Python Software Foundation, https://www.python.org).

### 1.1. Contact Structure

On weekdays students go to school and we explicitly model contacts from 7:30 AM to 3:30 PM, with contacts modeled to reflect COVID-19 precautions to limit mixing across classrooms. We model contacts in half-hour intervals in three different school contexts: (i) students commuting to and from school on the bus, (ii) classrooms, and (iii) breaks with school-wide interactions (Appendix Table 1). We did not include more elaborate contact patterns that have been analyzed in other studies (*1*; A. Bilinski, unpub. Data, https://doi.org/10.1101/2021.05.12.21257131).

We assumed that adults working at schools can be infected by students, but not by other adults. Given the relatively small number of adults working in schools and their presumed higher levels of compliance with precautionary measures, incorporating adult-to-adult transmission would only slightly increase the transmission rate.

### Classroom

Each classroom is composed of 20 students and a single teacher. Students and teachers are assigned to a classroom at the beginning of the simulation and remain in the same room throughout the simulation. Therefore, each student interacts with 19 other students, as well as the same teacher, while in the classroom. Students spend 6 hours a day in the classroom.

### Bus

All students commute to and from school by taking a bus. Each bus transports the same 20 students throughout the simulation and is driven by the same driver, and students are randomly assigned to a bus, independently of the classroom to which they belong. Therefore, each student interacts with 19 other students as well as the same driver while on the bus. Students spend 1 hour on the bus each day, half an hour at the beginning and at the end of the day.

### Break

During the noon break students interact with other students throughout the school in cliques of 10 students; in addition, each student interacts with two adults randomly selected from the teachers and staff. The cliques of students, as well the two adults each student interacts with, are randomly determined independently of classrooms and buses, and remain constant throughout the simulation. Therefore, each student interacts with 9 other students as well as two adults while on break. Students spend 1 hour on break each day (Appendix Table 1).

Outside of school, students interact with the adults in their households. The number of adults in each student's household is determined according to data collected by the US Census Bureau regarding living arrangements of children under age 18 (*2*). For simplicity, we do not explicitly model siblings within households who attend the same school. Household transmission between such siblings can amplify school-based outbreaks if siblings who are infected at home return to school while infectious. However, the household attack rate of 16.6% (*3*), and the limited number of sibling pairs in different classrooms (*4*), would make such occurrences relatively small. In addition, the baseline quarantine strategy of quarantining an entire individual's household upon a positive test would prevent some of those sibling infections from spreading further in school.

Rather than explicitly modeling contacts within households, we use a published estimate of the household attack rate of COVID-19 and assumed infected individuals, whether adults or children, symptomatic or not, transmit the disease to 16.6% of their susceptible household (*3*). The exact time of infection is determined randomly based on the relative infectiousness of an individual through time (details below).

All interactions with the broader community are abstracted and included through a single daily community incidence value that is kept constant throughout the simulation. We determined the number of new infections due to community interactions through a binomial process. For instance, if we denote the daily community incidence of COVID-19 by $p$, e.g., $p = 70$ new daily cases per 100,000 population, then the number of newly infected individuals in a group with $N$ individuals is sampled from a binomial distribution as $Binomial(N,p)$.

To account for time spent in school, the daily incidence of new cases among members of the school environment on weekdays is half that of the community, but incidence is the same on weekends.

### 1.2. COVID-19 Natural History

The natural history of COVID-19 is modeled according to the diagram shown in Appendix Figure 1. Infected individuals move to the exposed compartment (E) before progressing to either a presymptomatic (PY) or pre-asymptomatic (PA) compartment, from which they then move to the symptomatic infectious (IY) and asymptomatic infectious (IA) compartments, respectively. From there all infected individuals recover (R) and become immune to the disease. The transition times from one compartment to another are determined at the time of infection and follow the probability distributions listed in Appendix Table 2.

The infectiousness of all individuals varies through time, and given the average sojourn times in each compartment, the infectiousness profile follows a gamma distribution with shape and scale parameters of 2.0 and 1.55, shifted left by 2.3 days. The resulting profile is shown in blue in Appendix Figure 2, and based on the time of its maximum value (mode) this results in peak infectiousness of ≈0.7 day before symptom onset, with 45% of total infectiousness for an individual occurring in the pre-(a)symptomatic compartment, following the results from He et al. (*5*). The curve is normalized so the average infectiousness is 1.0.

As the time spent in each compartment by different individuals is randomly sampled from the probability distributions listed in Appendix Table 2, the infectiousness profile in blue in Appendix Figure 2 is adjusted for the specific time spent by an individual in the pre-(a)symptomatic and (a)symptomatic compartments using the standard times of symptom onset (or transition to infectious asymptomatic compartment), start of infectiousness (transition of pre-(a)symptomatic compartment), and recovery as the control points. For instance, individual 1 shown in green in Appendix Figure 2 spends less time in the pre-symptomatic compartment than average so the green curve before time 0 has the same shape as the blue one, but is compacted from 2.3 days to 1.8 days. Then individual 1 spends more time in the symptomatic compartment than average, so the green curve after time 0 has the same shape as the blue curve, but it is stretched to 10 days.

In addition, individuals who remain asymptomatic are assumed to be two-thirds as infectious as symptomatic persons at any given point (*6*), with 20% of children and 57% of adults becoming symptomatic (*7*,*8*).

### 1.3. Infection Events Modeling

When an infected individual indexed by $j$ interacts with a susceptible individual indexed by $k$ at time $t$ in school for one time step of half an hour, the probability that $j$ infects $k$ is given by:

$$q_t = \beta \cdot \omega_j \cdot i_j(t)$$

where $\omega_j$ = 1.0 if individual $j$ is symptomatic or pre-symptomatic and $\omega_j$ = 2/3 if individual $j$ is asymptomatic or pre-asymptomatic; here, $i_j(t)$ represents the relative infectiousness of individual $j$ at time $t$ as shown in Appendix Figure 2, and $\beta$ is an input parameter that is selected so that the unmitigated reproduction number $R_0$ of children in school is equal to a chosen input. It represents the probability that a symptomatic individual with relative infectiousness of 1.0 at time $t$ infects a susceptible contact in a half-hour interval.

We use the following to determine $\beta$:

$d_I = d_P + d_S$ the total infectious period in days, which is the sum of the pre-(a)symptomatic and (a)symptomatic periods

$\tau$ proportion of symptomatic students

$h_x$, $C_x$ the number of hours spent in school context $x$ (of the 3 contexts detailed above) and the number of contacts in that context

$\omega_a = 2/3$, $\omega_s = 1$ scaling factor of the relative infectiousness of asymptomatic and symptomatic individuals through their infection $\Delta t = 1/2$-hour single time step duration in the simulations

We can calculate the desired $R_0$ in school as the sum of $R_0$ in the three different school contexts: $R_0 = R_0(\text{class}) + R_0(\text{bus}) + R_0(\text{break})$.

The basic reproduction number in a given place is simply the probability of infecting a given contact multiplied by the number of contacts: $R_0(x) = C_x \cdot p(x)$, where $p(x)$ represents the probability of infecting a single individual contact in context $x$ over the individual's entire infectious period. Then:

$$R_0 = p(class) \cdot C_{class} + p(bus) \cdot C_{bus} + p(break) \cdot C_{break}$$

$$R_0 = p(class) \cdot [C_{class} + \frac{1}{h_{class}}(C_{bus} \cdot h_{bus} + C_{break} \cdot h_{break})]$$

where we make the simplification that the probability of infecting an individual is directly proportional to the total time spent in contact with them.

We now only need to calculate the probability of infecting an individual who is a classroom contact, $p(class)$. This probability is averaged over symptomatic and asymptomatic individuals. To simplify calculations, we calculated the probability that a symptomatic individual would infect another person in their classroom, $p(class)$, with subscript $s$ to denote symptomatic, which is given by:

$$p_s(class) \cdot [\tau + \omega_s + (1 - \tau) \cdot \omega_a)] = p(class).$$

We can then write the relationship between the probability of a symptomatic infectious individual infecting a contact at some point in time, with the probability of infection at each time step in the simulation via:

$$1 - p_s(class) = \prod_{t=t_0}^{T}(1 - q_t)$$

By definition, for a symptomatic individual we have $q_t = \beta \cdot i_j(t)$, which depends on $t$ through the individual's relative infectiousness. We simplify this by $q_t = \beta$ using the fact that $i_j(t)$ is constructed to have an average value of 1. As a result, we have the following:

$$1 - p_s(class) = \prod_{t=t_0}^{T} (1 - \beta) = (1 - \beta)^N$$

where $N$ represents the average number of time steps that an infected individual has with other contacts in their classroom while infectious:

$$N = \frac{h_{class}}{\Delta t} \cdot d_I \cdot \frac{5}{7}$$

and we use 5/7 to represent the fact that school days occur on weekdays only.

Finally, we obtain

$$\beta = 1 - [1 - p_s(class)]^{\frac{1}{N}}.$$

#### 1.4. Testing and Isolation

In all scenarios, we assumed that 90% of symptomatic individuals will seek testing once symptoms occur. The exact time after symptom onset at which individuals seek testing is random and is sampled from a triangular distribution with an average of 1 day, a lower bound of 0.5 day, and an upper bound of 1.5 day. We assume that the tests that symptomatic individuals use are perfect, and separate from the surveillance testing budget. In addition, 2 hours after getting tested, the symptomatic individual starts their isolation, and if applicable their contacts quarantine themselves at the same time. For surveillance testing the results are assumed to be instantaneous. The infected individual then starts their isolation immediately, and their contacts quarantine themselves immediately as well. In the baseline scenarios all isolations and quarantines last 14 days, according to the Austin Independent School District (AISD) policy during the 2020–2021 school year (*9*).

In our simulation, surveillance tests are all done on Mondays at 8 AM, right after students take the bus to school and before the first class. If students are isolated or quarantined for 2 weeks, they come back to school on the second Monday after testing positive.

In addition, surveillance tests are allocated across classrooms every week. For instance, if 50% of a school's students are tested every week, then 50% of the students in each classroom are

tested each week, according to a defined schedule so that every student will be tested every other week. This method of test allocation within a school performed better in our experiments than randomly selecting the students for testing, or testing entire classrooms some weeks while no student is tested in other classrooms.

We assumed that individuals strictly quarantine themselves, so that they cannot be infected with contacts from the broader community. However, in this case, students could still be infected by one of their household members.

In our base-case scenario, we assumed that surveillance tests were perfect, but we ran some sensitivity analysis relaxing this assumption. We ran some scenarios where the tests had a sensitivity of 95% for symptomatic individuals, 80% for pre-(a)symptomatic and asymptomatic individuals, and a 99% specificity, which corresponds to pre-Delta estimates published for the Abbott BinaxNOW tests (*10*,*11*). Parameters are provided in Appendix Table 2.

## 2. Optimization Model

We optimize the allocation of tests across a set of schools to minimize a chosen risk metric. The results shown in the main text seek to minimize the maximum risk across schools and the associated model is presented in the following section. We have explored other objective functions, and we show the corresponding model formulations in this section as well as the resulting allocations as a sensitivity analysis further below.

As part of our optimization model formulation, we use the preprocessed results from the simulation model above as inputs, so the optimization part of the framework is run independently and subsequently to the disease transmission model.

### 2.1. Notation

Set and indices: $s \in S$ set of schools in the system.

Parameters: $N_s$ number of students in school $s$; $B$ testing budget, expressed as the proportion of students in the entire system that can be tested weekly.

Variables: $t_s$ proportion of students tested each week in school s; $I_s(t_s)$ proportion of students in school s infected on-campus over the horizon under testing regime $t_s$.

The decision variable $t_s$ represents the testing regimen in a school and is expressed as the proportion of the school's students tested each week. For example, $t_s = 50\%$ means that every week 50% of the school's students are screened so that, on average, students get tested every other week, while with $t_s = 33\%$ students are tested every 3 weeks on average. Evaluating $I_s(t_s)$ requires running the simulation model of disease transmission that we sketch above for 300 simulations, given a specific value of $t_s$.

### 2.2. Model

The objective of the optimization model is to minimize the maximum risk experienced by any school in the system subject to two constraints.

First, we cannot allocate more tests than are available in the budget:

$$\sum_{s \in S} N_s \cdot t_s \leq B \cdot \sum_{s \in S} N_s \qquad [C1]$$

Second, we limit testing to a weekly frequency in each school:

$$0 \leq t_s \leq 1, \forall s \in S \qquad [C2]$$

The risk metric we aim to minimize is the average of (i) the on-campus expected infection rate across simulations $\mathrm{E}\{I_s(t_s)\}$, and (ii) the conditional value-at-risk (CVaR) of the infection rate at a level $\beta = 90\%$, $CVaR_{90}\{I_s(t_s)\}$. Therefore, the risk for school $s$ given a testing regimen $t_s$ is

$$R_s(t_s) = \frac{1}{2}[\mathbb{E}\{I_s(t_s)\} + CVaR_{90}\{I_s(t_s)\}].$$

CVaR, also called expected shortfall, is a widely used risk measure in stochastic optimization, thanks to its coherence properties, ease of interpretation, and computational tractability (*13*,*14*). Consider a random variable, X, that we would like to keep "small," such as the proportion of a population that is infected with COVID-19. CVaR is the conditional expectation given that X exceeds its $\beta$-level quantile. Thus, in our case, with $\beta = 0.90$, $CVaR_{90}\{I_s(t_s)\}$ computes the conditional expectation of the worst 10% of the outcomes, or the average proportion of infected students at a school, when restricting that average to the 30 simulated scenarios out of 300 with the largest proportion of infections. Hence, including this term helps determine a test-allocation strategy that controls tail risk. Formally, for a random

variable X with cumulative distribution function $F(x)$, value-at-risk (VaR) and CVaR are defined as follows:

$$VaR_\beta(X) = \inf\{x : F(x) \geq \beta\} = F^{-1}(\beta)$$
$$CVaR_\beta(X) = \mathbb{E}\{X | X \geq VaR_\beta(X)\}.$$

Then the optimization problem can be expressed as the following:

$$\min_{t_s} \max_{s \in S} R_s(t_s)$$
$$s.t. [C1], [C2]$$

Put together and reformulated, this gives us the following optimization model, where the decision variables are the various amounts of testing $t_s$ in each school:

$$(P) \quad \begin{aligned} \min \quad & z \\ s.t. \quad & \frac{1}{2}\left[\mathbb{E}\{I_s(t_s)\} + CVaR_{90}\{I_s(t_s)\}\right] \leq z, \quad \forall s \in S \\ & \sum_{s \in S} N_s \cdot t_s \leq B \cdot \sum_{s \in S} N_s \\ & 0 \leq t_s \leq 1, \quad \forall s \in S \end{aligned}$$

Due to the nonlinear, and effectively black-box, nature of the functions $I_s(t_s)$ with respect to $t_s$ and the fact that we summarize the risk of each school using the infection rate's expected value and CVaR, this is a nonlinear black-box continuous optimization problem that can be solved with standard solvers. We used the COBYLA method implemented in SciPy/Python to solve the problem (*15*,*16*).

Our optimization model is nonlinear and nonconvex but has two key properties that allow us to readily check whether a solution is globally optimal. First, the risk function associated with each school

$$R_s(t_s) = \frac{1}{2}[\mathbb{E}\{I_s(t_s)\} + CVaR_{90}\{I_s(t_s)\}]$$

is a decreasing function of the allocation $t_s$. Second, we are solving a continuous minimax problem in which we are focused on the school with the worst-case risk. Thus, we can first allocate resources to the worst-case school to decrease its risk to that of the school with the second-highest risk, and repeat this scheme until the budget is exhausted, dealing with obvious edge cases. This allows us to verify that the solution found via COBYLA is indeed a globally optimal solution.

Testing cannot be more frequent than weekly, i.e., $t_s \leq 1$. When solving problem ($P$), we may observe that some schools, indexed by say, $s \in S'$, are allocated enough tests for weekly testing. To help reduce the dimension and aid the solver in its search, we can fix the corresponding decision variables of the schools with weekly testing $t_s$, $s \in S'$, to 1, and rerun COBYLA to optimize the allocation for the remaining schools, excluding those schools from the first constraint in ($P$). The problem then becomes the following:

$$(P') \quad \begin{aligned} \min \quad & z \\ s.t. \quad & \frac{1}{2}\left[\mathbb{E}\{I_s(t_s)\} + CVaR_{90}\{I_s(t_s)\}\right] \leq z, \quad \forall s \in S \backslash S' \\ & \sum_{s \in S} N_s \cdot t_s \leq B \cdot \sum_{s \in S} N_s \\ & 0 \leq t_s \leq 1, \quad \forall s \in S \backslash S' \\ & t_s = 1, \quad \forall s \in S' \end{aligned}$$

After sketching two alternative formulations, we describe another means by which we help the optimization algorithm in terms of preprocessing output from the simulation model.

#### 2.3. Alternative Objective Functions

Below we show the optimization model when minimizing different objective functions. In Appendix Figure 15, we show the resulting optimal allocations from solving those problems for the hypothetical school system.

Varying CVaR Level and Relative Weight

Two straightforward modifications to the original optimization problem are to change the level $\beta$ of CVaR as well as the respective weights of CVaR and of the expectation in the risk level of each school. The problem is easily modified, using some value, $w \in [0,1]$ for the weight of CVaR, we have the following:

$$(P_{w,\beta}) \quad \begin{aligned} \min \quad & z \\ s.t. \quad & (1-w) \cdot \mathbb{E}\{I_s(t_s)\} + w \cdot CVaR_{\beta}\{I_s(t_s)\} \leq z, \quad \forall s \in S \\ & \sum_{s \in S} N_s \cdot t_s \leq B \cdot \sum_{s \in S} N_s \\ & 0 \leq t_s \leq 1, \quad \forall s \in S \end{aligned}$$

By setting $w$ to 0 or 1 we can focus either on the expected infection rate or on the CVaR of infection rates respectively, thus ignoring tail risk entirely or focusing solely on it.

Total Expectation

A different approach is to try to minimize total infections occurring across the entire system rather than trying to minimize the risk for the highest-risk school, as in the main text. Then the size of different schools, through the number of students $N_s$, directly impacts the objective function. The problem is formulated as follows:

$$
(P_{total}) \quad \begin{aligned} \min \quad & \sum_{s \in S} N_s \cdot \mathbb{E}\{I_s(t_s)\} \\ s.t. \quad & \sum_{s \in S} N_s \cdot t_s \leq B \cdot \sum_{s \in S} N_s \\ & 0 \leq t_s \leq 1, \quad \forall s \in S \end{aligned}
$$

**2.4. Inputs Preprocessing**

To run the optimization model, we need to preprocess the results from the transmission model to smooth out some of the stochasticity of the simulations. We do this in two steps.

First, we take the results from the 300 simulations for a single parameter set, which corresponds to a specific school with a certain testing frequency, and we fit a gamma distribution to the proportion of students infected on-campus (Appendix Figure 3, panel A). This ensures that the risk metrics we calculate are not biased by single simulations and that the CVaR metric stays continuous with respect to the exact level $\beta$ chosen.

Second, using those fitted gamma distributions, we calculate the risk for all schools for each of the 21 testing amounts simulated, from no students tested to all students tested weekly, in 5% increments. This produces the blue dots in Appendix Figure 3, panel B. We then fit a non-increasing curve to these dots, specifically we fit a cubic curve up to the point where 75% of students are tested weekly and then a linear curve. This piecewise definition of the curve helps us keep the number of parameters necessary for fitting low enough to have a parsimonious model while having a good fit.

This process ensures that the risk level of a school decreases as the testing frequency increases, which might not always be the case in our simulations due to stochasticity, especially when the number of infections is already low. Additionally, explicitly pre-calculating the risk level of a school as a function of the testing frequency ensures rapid execution of the optimization routine because the COBYLA algorithm might require a large number of iterations when the risk level of each school must be evaluated.

## 3. AISD Details

The list of high schools from the Austin Independent School District (AISD) included in our analysis is given in Appendix Table 3 along with the number of students modeled in our simulations (*17*). The number of students in each school is based on enrollment reports from the Texas Education Agency (TEA) multiplied by the reported in-person attendance of 6% in AISD high schools at the beginning of January 2021 (*18*,*19*). We rounded the numbers of students to get classes of 20 students and calculated the number of adults working in the school by scaling the numbers given in Appendix Table 2 to the numbers of students in each high school.

Figure 3 panels A, B in the main text show the catchment area of each of the 11 high schools along with the relative community incidence in each of those schools, as well as the basic reproductive number ($R_0$) assigned to each of those schools for the part of the analysis where $R_0$ varies across campuses. The methodology used to determine the community incidence of each school catchment area is detailed in the next section.

To analyze the impact of schools having different transmission rates we assigned each school an $R_0$ value, but those values are rough approximations based on limited data and do not necessarily represent what actually happened in the schools during the academic year 2020–2021.

To assign $R_0$ to each school, we used the reported COVID-19 cases in each AISD school from the district's public dashboard on March 8, 2021 (*20*), as well as the total enrollment and staff data per school used by TEA for allocation of tests in the context of the K–12 COVID-19 testing project (*21*).

We then regressed the number of reported cases $y$ on the total school population $x$ and obtained a best fit line of the form: $y = y_0 + \alpha \cdot x = 5.82 + 0.0095 \cdot x$ with $R^2 = 0.44$, as shown in Appendix Figure 4.

We then assigned an $R_0$ in each school proportional to the square root of the ratio of reported cases $y_i$ to predicted cases:

$$\hat{y}_i = y_0 + \alpha \cdot x_i$$

with a base value of $R_0 = 1$. For a school that reported $y_i$ cases with a total population $x_i$ we assigned the following:

$$R_{0,i} = \sqrt{\frac{y_i}{y_0 + \alpha \cdot x_i}}.$$

The values assigned to each school are listed in Appendix Table 3.

#### 3.1. COVID-19 Incidence in School Catchment Areas

To calculate the relative community incidence of COVID-19 cases for each school we combine catchment area information from AISD and COVID-19 burden data in the Austin metropolitan statistical area (MSA).

We first obtain the cumulative COVID-19 hospitalization rate, $h_j$, per 100,000 persons for each postal code $j$ in the Austin MSA from March 2020–January 11, 2021 (*22*); then, we calculate the average hospitalization rate in the metropolitan area, $h_{MSA}$. From there we computed the relative burden experienced by each postal code $j$ as follows:

$$r_j = \frac{h_j}{h_{MSA}}.$$

Next, we estimated the proportion of students in each school $i$ coming from postal code $j$ ($p_{ij}$) by using the proportion of school $i$'s catchment area located in postal code $j$ as a proxy. To do so, we use GIS data from AISD and Austin MSA (*23*,*24*).

We denote by $A_i$ the area of the catchment area of school $i$, the area covered by postal (i.e., ZIP) code $j$ ($Z_j$), and $A_i \cap Z_j$ the area of the intersection of the two. We then have,

$$p_{ij} = \frac{A_i \bigcap Z_j}{A_i}$$

Combining the two quantities we then estimate the relative community incidence $s_i$ of each school as the weighted average of the postal codes relative burden via the following:

$$s_i = \sum_j p_{ij} r_j \ .$$

#### 3.2. Additional Results

Appendix Table 4 gives the cumulative on-campus attack rate in each school for three allocation strategies: no testing, prorated allocation of 14-day total testing capacity, and optimal allocation of 14-day prorated total testing capacity. The results are given for the scenarios in

which all schools have different transmission rates (first three substantive columns), and when they all have the same transmission rate ($R_0$ of 1.0, last three columns). The prorated and optimal allocations when schools have different transmission rates correspond to the results shown in Figure 4 panels A, B.

The additional results below analyzing the 11 high schools in AISD assume rapid tests with a sensitivity of 95% for symptomatic individuals, 80% for asymptomatic people, and a 99% specificity, based on reported estimates of the Abbott BinaxNOW antigen tests (*10*,*11*).

Appendix Figure 5 shows results similar to those shown in Figure 4 panels A, B in the main text, but here the transmission rate in all schools is identical and equal to 1.0. The blue bars showing the optimal allocation in the left panel correspond to the diamonds shown in Figure 4, panel A (main text). Appendix Figure 6 shows results similar to those shown in Figure 5 (main text), except that here the schools have the same transmission risk of $R_0$ = 1.0.

Appendix Figure 7 shows the risk level of each school under an optimal allocation when the on-campus transmission rate used as input to the optimization problem is different from the actual transmission rate of each school. Specifically, we derived an optimal allocation assuming all schools have the same on-campus transmission rate $R_0$ of 1.0 when schools actually have different on-campus transmission rates. The gray dots correspond to the resulting risk levels under that allocation, while the blue dots correspond to the risk level achieved when using the correct transmission rate to derive the optimal allocation, and the orange dots correspond to a pro rata allocation. The risk of most schools ends up being similar under the two optimal allocations, with the allocation derived using the wrong transmission rates typically resulting with in a risk level that is closer to the optimized risk level than that of the pro rata allocation, with the exception of schools H and I for which the pro rata allocation happens to be very close to optimal in that scenario.

## 4. Toy System Results

Appendix Table 5 below contains the details of the numbers shown in Figure 1, panel A in the main text. It gives the expected proportion of students infected on-campus for different quarantine strategies, testing frequencies, and in-school $R_0$. Appendix Table 6 contains the

details of the numbers shown in Figure 2, panel A in the main text. It gives the proportion of school days students either miss school, infected or not, or are at school while infected.

## 5. Toy System Optimization

We show the results of the optimal test allocation on a toy system of hypothetical schools (Appendix Figures 8, 9, 14). There are six schools of 500 students each in this system, three with a low daily community incidence of new cases (35 per 100,000) and three with a high daily community incidence (70 per 100,000). In each of the two groups the schools have different in-school transmission rates, low (unmitigated $R_0$ = 1.0), moderate (unmitigated $R_0$ = 1.5), and high (unmitigated $R_0$ = 2.0).

Appendix Figure 8 shows the number of averted infections through surveillance testing with a 2-week testing frequency budget, compared to no surveillance testing, for both a prorated allocation and an optimal allocation. Using the distributions of on-campus infections for a school under the scenario without testing, and under a scenario with testing, we can calculate the number of averted infections through testing using inversion sampling. We denote by $F_{no\ testing}$ and $F_{testing}$ the cumulative distribution functions (CDF) for the number of on-campus infections of the scenarios without testing and with testing, respectively. We then generate 300 random numbers $U_j, j \in [1, 300]$, uniformly distributed between 0 and 1, and we calculate the number of averted infections as follows:

$$A_j = F^{-1}_{no\,testing}(U_j) - F^{-1}_{testing}(U_j), j \in [1, 300].$$

As we move from no testing to a prorated allocation, we decrease the risk of all schools, and when we move from the prorated allocation to an optimal allocation, the performance of the three lowest-risk schools worsens slightly, i.e., the low-low, low-moderate, and high-low schools (incidence-transmission pairs). Meanwhile the number of cases averted at the remaining three schools grows significantly; hence, the total system risk decreases in an optimal allocation.

Appendix Figure 9 shows the optimal allocation of tests to all schools under different testing budgets, with the 14 days column corresponding to the allocation that yields the distributions of averted cases shown in Appendix Figure 8. No matter the testing frequency, under an optimal allocation the testing capacity is diverted from the same three low-risk schools

to the higher-risk schools. The low-low school receives the fewest tests under all budgets, while the high-high school receives the most. For instance, for a budget that would allow us to test all students in the system every 10 days on average, it would be optimal to test all students in the high-high school every week, while it would only be necessary to test students in the low-low school every 4 weeks to achieve the same overall level of risk. As a result, the risk profiles of all schools are very similar under the optimal allocation below that shows the distribution of on-campus infections for all schools under prorated and optimal allocations (Appendix Figure 14).

## 6. Sensitivity Analysis

### 6.1. Quarantine Strategy and Imperfect Surveillance Tests

It is now widely accepted that antigen tests for COVID-19 are effective at detecting infectious people, but there have been debates regarding their true performance (*25*). As such, we ran some sensitivity analyses using imperfect surveillance tests. Specifically, we used a test sensitivity of 95% for symptomatic individuals, 80% for asymptomatic (including pre-symptomatic) people, and we used a specificity of 99%, corresponding to published estimates of the Abbott BinaxNOW tests (*10*,*11*). Appendix Figure 10 shows results similar to those shown in Figure 1A and 1B (main text), the proportion of students infected in schools under different testing frequencies, for the same two quarantine strategies considered in Figure 1 (main text), as well as a scenario using imperfect tests and classroom quarantines.

### 6.2. School Days Missed

In Figure 2, panel A (main text) we detail the proportion of school days missed for a school with low community incidence and moderate transmission risk, for two quarantine strategies. In Appendix Figure 11, we show analogous results for the other hypothetical schools considered in our system, as well as the case in which imperfect tests are used.

The results are qualitatively similar across schools. We notice that holding other factors constant, a higher community incidence leads to more school days missed as more introductions of the disease in the school lead to more students being quarantined.

The bottom panels in each figure consider the case in which entire classrooms are quarantined while using imperfect tests with 99% specificity and with 80% and 95% sensitivity for asymptomatic and symptomatic individuals, respectively. While these tests reduce cases to a

similar extent as perfect tests (see Appendix Figure 10) the false positives (students testing positive despite not being infected) lead to students missing more school time while healthy, with school days missed increasing with the testing frequency.

We can also see that when the transmission risk is high, increasing the testing frequency does not lead to more school days missed when quarantining entire classrooms. Indeed, when the transmission risk is low enough some of the infected individuals found through surveillance testing would not infect anyone else, but entire classrooms are still quarantined, so that more frequent testing leads to more missed days. On the other hand, when the transmission rate is high more frequent testing allows us to find infected individuals earlier, before more infections occur, thus breaking transmission chains. This effect is also seen by looking at the proportion of days in school while infected when only households are quarantined: when the transmission risk is low that proportion decreases slowly as testing increases while it decreases dramatically as testing frequency increases when transmission risk is high.

### 6.3. Quarantine Duration and Contacts Quarantined

While many school districts have used a policy of quarantining contacts of a person testing positive for COVID-19 for 14 days, the Centers for Disease Control and Prevention (CDC) has continually been updating its interim guidance throughout the pandemic, saying in February 2021, that 10 days of isolation were sufficient in most cases, and in January 2022, it further shortened the isolation period to 5 days (*26*). We evaluated the impact of shorter isolation periods, either 7 days or 10 days, and compared it with our baseline assumption of a 14-day isolation period. Appendix Figure 12 shows the tradeoff between reducing infections and minimizing missed school days for different quarantine strategies at various testing frequencies for schools with a moderate transmission risk.

The different quarantine strategies are represented by different marker types and darker colors indicate more frequent surveillance testing. While quarantining entire classrooms for longer periods reduces infections more than shorter quarantines, it also leads to more school days missed for students. When testing is frequent enough, nearing weekly testing, longer quarantines are only marginally more effective at preventing infections (points closer to the x-axis) at the expense of more school days missed (points further from the y-axis).

### 6.4. Total Testing Budget

One way to visualize the allocation of tests across a set of schools is to graph the risk level of all schools as a function of the testing frequency in the same graph. Appendix Figure 13 shows the risk level of each of the six hypothetical schools in the system we consider, as we move from no surveillance testing to weekly testing. The risk of each school is again defined as the average of the expected on-campus infection rate and CVaR.

As the testing frequency increases the risk of each school decreases. The objective of our optimization is to find the lowest horizontal line on the graph such that the testing budget is respected. A horizontal line corresponds to all schools having the same risk, unless some school has reached the maximum testing frequency of 7 days, or a school does not need any testing to reach the same risk level as the other schools.

The left y-axis represents each school's risk, while the right y-axis represents the necessary budget required to achieve the corresponding maximum risk across schools. We can see that the lower the risk across schools becomes, the more expensive it becomes to further decrease it; to achieve a similar decrease requires much more frequent testing, so many more tests every week.

Given a certain budget we can draw a horizontal line at the corresponding point on the right axis and the intersection of this line with each of the schools' curves gives us the optimal allocation that minimizes the maximum risk across schools.

We can then verify that the full risk profiles of the six schools are very similar under the optimal allocation for the two budgets shown by the horizontal lines above. Appendix Figure 14 shows the distribution of on-campus infections in all the schools for three testing strategies. The no testing strategy shows the risk of each school without testing, the pro rata allocation shows the risk of all schools under the suboptimal allocation in which all schools receive the same number of tests, while under the optimal allocation all schools have a similar risk profile, and the total system risk is lowest.

### 6.5. Objective Function Optimized

In Appendix Figure 15 we show the sensitivity of the optimal allocation of tests in each school as we parametrically vary the objective function. We consider two testing budgets, first a budget in which all students can be tested every 4 weeks on average, and then every 2 weeks.

In the left panels we fix the weight of the CVaR term to 50%, and thus we have equal weight for the expected value of the infection rate when defining the risk of each school, $R(s)$, and we vary the CVaR level $\beta$. That is we solve the problem ($P_{50\%\beta}$) where $\beta$ goes from 5% to 95%. In the middle panel we fix $\beta = 90\%$ and we solve ($P_{w,90\%}$) for $w$ increasing from 0% to 100%, i.e., increasing the weight on the CVaR term. In the right panels we solve the problem ($P_{total}$), which aims to minimize the total system-wide expected infections.

When we only have a budget to test students every 4 weeks the impact of $w$ and $\beta$ on the solution of ($P_{w\beta}$) is limited, with only the school with the pair of low community incidence and moderate $R_0$ being allocated marginally more tests as $w$ and $\beta$ increase.

However, when we have a budget to test students every 2 weeks, we see much more variation in the allocation. This comes from the fact that with that budget the school with the pair of high community incidence and high $R_0$ is being allocated nearly enough tests to test weekly. As can be seen in the earlier Appendix Figure 13, the risk of that school does not change much as testing changes from every 9 days to every 7 days, so that small changes in $w$ or $\beta$ can impact the school risk enough to then cause large changes in the allocations to the other schools. While the changes in allocation for the school with high community incidence and high $R_0$ can seem large for small changes in the school risk parameters, the allocations to the other schools do not change drastically.

Lastly, in the right panels we see that when minimizing the total infections across all schools the same schools receive more tests than under a pro rata allocation, but there tends to be smaller differences in the number of tests allocated across schools as all schools receive a share closer to their pro rata allocation.

**References**

1. Bilinski A, Salomon JA, Giardina J, Ciaranello A, Fitzpatrick MC. Passing the Test: A Model-Based Analysis of Safe School-Reopening Strategies. [Internet]. Ann Intern Med. 2021;174:1090–100. PubMed https://doi.org/10.7326/M21-0600

2. US Census Bureau. Living arrangements of children under age 18 [cited 2021 Apr 14]. https://www.census.gov/library/visualizations/2016/comm/cb16-192_living_arrangements.html

3. Madewell ZJ, Yang Y, Longini IM Jr, Halloran ME, Dean NE. Household transmission of SARS-CoV-2: a systematic review and meta-analysis. JAMA Netw Open. 2020;3:e2031756. PubMed https://doi.org/10.1001/jamanetworkopen.2020.31756

4. Dur U, Morrill T, Phan W. Family ties: school assignment with siblings [cited 12 Sep 2022]. https://thayermorrill.wordpress.ncsu.edu/files/2019/06/FamilyTies.pdf

5. He X, Lau EHY, Wu P, Deng X, Wang J, Hao X, et al. Temporal dynamics in viral shedding and transmissibility of COVID-19. Nat Med. 2020;26:672–5. PubMed https://doi.org/10.1038/s41591-020-0869-5

6. He D, Zhao S, Lin Q, Zhuang Z, Cao P, Wang MH, et al. The relative transmissibility of asymptomatic COVID-19 infections among close contacts. Int J Infect Dis. 2020;94:145–7. PubMed https://doi.org/10.1016/j.ijid.2020.04.034

7. Gudbjartsson DF, Helgason A, Jonsson H, Magnusson OT, Melsted P, Norddahl GL, et al. Spread of SARS-CoV-2 in the Icelandic Population. N Engl J Med. 2020;382:2302–15. PubMed https://doi.org/10.1056/NEJMoa2006100

8. Davies NG, Klepac P, Liu Y, Prem K, Jit M, Eggo RM; CMMID COVID-19 working group. Age-dependent effects in the transmission and control of COVID-19 epidemics. Nat Med. 2020;26:1205–11. PubMed https://doi.org/10.1038/s41591-020-0962-9

9. Austin Independent School District. Health FAQs [cited 2021 Apr 14]. https://www.austinisd.org/openforlearning/healthsafety/health-faqs

10. US Food and Drug Administration. Abbott BinaxNOW COVID-19 Ag CARD instructions for use and characteristics [cited 2021 Apr 19]. https://www.fda.gov/media/141570/download

11. Pollock NR, Jacobs JR, Tran K, Cranston AE, Smith S, O'Kane CY, et al. Performance and implementation evaluation of the Abbott BinaxNOW Rapid Antigen Test in a high-throughput drive-through community testing site in Massachusetts. J Clin Microbiol. 2021;59:e00083-21. PubMed https://doi.org/10.1128/JCM.00083-21

12. Zhang J, Litvinova M, Wang W, Wang Y, Deng X, Chen X, et al. Evolving epidemiology and transmission dynamics of coronavirus disease 2019 outside Hubei province, China: a descriptive and modelling study. Lancet Infect Dis. 2020;20:793–802. PubMed https://doi.org/10.1016/S1473-3099(20)30230-9

13. Acerbi C, Tasche D. Expected shortfall: a natural coherent alternative to value at risk. Econ Notes. 2002;31:379–88. https://doi.org/10.1111/1468-0300.00091

14. Rockafellar RT, Uryasev S. Optimization of conditional value-at-risk. Journal of Risk. 2000;2:21–41. https://www.ise.ufl.edu/uryasev/files/2011/11/CVaR1_JOR.pdf

15. Powell MJD. On trust region methods for unconstrained minimization without derivatives. Math Program. 2003;97:605–23. https://doi.org/10.1007/s10107-003-0430-6

16. Virtanen P, Gommers R, Oliphant TE, Haberland M, Reddy T, Cournapeau D, et al.; SciPy 1.0 Contributors. SciPy 1.0: fundamental algorithms for scientific computing in Python. Nat Methods. 2020;17:261–72. PubMed https://doi.org/10.1038/s41592-019-0686-2

17. Austin Independent School District. About our schools, high schools [cited 2021 Jun 16]. https://www.austinisd.org/schools/level/H

18. Texas Education Agency. Student enrollment reports [cited 2021 Apr 14]. https://rptsvr1.tea.texas.gov/adhocrpt/adste.html

19. KVUE. Austin ISD reports 2 weeks of declining cases; 18% of student population back to in-person learning 2021 [cited 2021 Apr 14]. https://www.kvue.com/article/news/education/schools/austin-isd-reports-2-weeks-of-declining-cases/269-1d56a09b-0b89-44b6-b37b-dbe7ffc7d3c2

20. Austin Independent School District. COVID-19 dashboard [cited 2021 Jun 17]. https://www.austinisd.org/dashboard

21. Texas Education Agency. Texas K–12 COVID-19 testing project [cited 2021 Apr 13]. https://tea.texas.gov/texas-schools/health-safety-discipline/covid/covid-19-support-public-health-orders#project

22. University of Texas at Austin COVID-19 Modeling Consortium. Heterogeneous burden of the COVID-19 pandemic in central Texas [cited 2021 Apr 25]. https://sites.cns.utexas.edu/sites/default/files/cid/files/austin_covid-19_spatial_burden_report.pdf

23. Austin Independent School District. School maps/GIS [cited 2021 Nov 13]. https://www.austinisd.org/planning-asset-management/school-maps-gis

24. City of Austin. GIS data [cited 2021 Nov 13]. https://austintexas.gov/department/gis-data

25. Mina MJ, Peto TE, García-Fiñana M, Semple MG, Buchan IE. Clarifying the evidence on SARS-CoV-2 antigen rapid tests in public health responses to COVID-19. Lancet. 2021;397:1425–7. PubMed https://doi.org/10.1016/S0140-6736(21)00425-6

26. US Centers for Disease Control and Prevention. Interim guidance on duration of isolation and precautions for adults with COVID-19 [cited 2021 Apr 19]. https://www.cdc.gov/coronavirus/2019-ncov/hcp/duration-isolation.html

**Appendix Table 1.** School day schedule used in a model for COVID-19 test allocation strategy to mitigate SARS-CoV-2 infections across school districts*

| Time | Context | Interactions |
|---|---|---|
| 7:30–8:00 AM | Bus | 19 students and 1 bus driver |
| 8:00 AM–12:00 PM | Classroom | 19 students and 1 teacher |
| 12:00–1:00 PM | Break | 9 students and 2 adults |
| 1:00–3:00 PM | Classroom | 19 students and 1 teacher |
| 3:00–3:30 PM. | Bus | 19 students and 1 bus driver |

*The interactions column indicates the number of contacts a single student has in each context. The individuals that a student interacts with are set at the beginning of a simulation and stay the same throughout.

**Appendix Table 2.** Transmission parameters used in a model for COVID-19 test allocation strategy to mitigate SARS-CoV-2 infections across school districts

| Parameter | Value | Source |
|---|---|---|
| Simulation duration | 10 weeks | |
| No. students | 500 | |
| No. adults in school | 25 teachers, 25 bus drivers, 16 staff | |
| $\tau$: proportion symptomatic | 20% of children, 57% of adults | Davies et al., Gudbjartsson et al. (*7*,*8*) |
| $w_A$: relative infectiousness of asymptomatic persons | 2/3 | He et al. (*6*) |
| $d_{inc}$: duration of incubation period | $d_{inc} \approx$ *Triangular*(3.2, 5.2, 7.2) (in days) | Zhang et al. (*12*) |
| $d_{pre}$: duration of pre-(a)symptomatic period | $d_{pre} \approx d_{inc}$ * *Triangular*(0.458, 0.558, 0.658) (average 2.9 d) | He et al. (*5*) |
| $d_S$: duration of (a)symptomatic period | $d_{inc} \approx$ *Triangular*(4, 8, 12) (in days) | He et al. (*5*) |
| Household attack rate | 16.6% | Madewell et al. (*3*) |
| $R_0$: unmitigated basic reproduction number of children | 1.0, 1.5, 2.0 | |
| $\beta$: probability of infection in a half-hour for individual with infectiousness of 1.0 | Fitted to $R_0$ | |
| Daily community incidence | 35 or 70 per 100,000 | Average high and low incidence estimated over several weeks in Austin, TX during winter 2020–2021 |

**Appendix Table 3.** List of high schools from Austin Independent School District included in a model for COVID-19 test allocation strategy to mitigate SARS-CoV-2 infections across school districts*

| School code | School name | No. students | Relative incidence† | School $R_0$ |
|---|---|---|---|---|
| A | LBJ | 60 | 1.95 | 1.41 |
| B | Navarro | 100 | 1.5 | 0.8 |
| C | Northeast Early College | 80 | 1.5 | 0.94 |
| D | Eastside Memorial | 40 | 1.49 | 0.87 |
| E | Travis | 80 | 1.18 | 0.84 |
| F | Crockett | 100 | 1.04 | 0.95 |
| G | Akins | 180 | 1.0 | 0.88 |
| H | Anderson | 140 | 0.7 | 1.1 |
| I | Austin | 140 | 0.58 | 1.26 |
| J | McCallum | 120 | 0.54 | 0.79 |
| K | Bowie | 180 | 0.37 | 0.98 |

*Includes assigned parameter values.
†Relative community incidence values are calculated by weighing the relative total attack rates of COVID-19 of the ZIP codes calculated in (*23*) using the proportion of students coming from each ZIP code as a weight. Schools are ordered according to their relative incidence.

**Appendix Table 4.** Median proportion of students infected in a model for COVID-19 test allocation strategy to mitigate SARS-CoV-2 infections across school districts*

| School code | Different transmission rates† | | | Same transmission rate‡ | | |
|---|---|---|---|---|---|---|
| | No testing | Prorated allocation | Optimal allocation | No testing | Prorated allocation | Optimal allocation |
| A | 14 (0.9–45.6) | 4.0 (0.1–22.9) | 2.2 (0–15) | 7.3 (0.4–34.2) | 2.7 (0.1–17) | 1.6 (0–10.8) |
| B | 5.3 (0.4–16.4) | 1.9 (0.1–9.6) | 1.4 (0–8.4) | 7.2 (0.6–25.5) | 2.6 (0.1–13.6) | 1.5 (0–8.8) |
| C | 6.3 (0.4–23.7) | 2.1 (0.1–11.9) | 1.3 (0–8.9) | 6.4 (0.4–23.5) | 2.4 (0.1–14) | 1.5 (0–10.2) |
| D | 3.6 (0–31.3) | 1.8 (0–13.7) | 1.0 (0–8.7) | 4.3 (0–33.7) | 2.1 (0–16.7) | 1.1 (0–9.5) |
| E | 3.9 (0.1–21.3) | 1.5 (0–9.5) | 1.3 (0–8.3) | 4.6 (0.1–26.4) | 1.7 (0–11.7) | 1.3 (0–9.5) |
| F | 4.3 (0.2–22.1) | 1.7 (0–10) | 1.4 (0–8.7) | 4.3 (0.1–23.6) | 1.8 (0–11) | 1.4 (0–9.3) |
| G | 4.3 (0.6–13.7) | 1.6 (0.1–7.7) | 2.0 (0.1–7.6) | 5.7 (0.6–17.4) | 2.1 (0.1–7.1) | 2.6 (0.2–8.8) |
| H | 4.6 (0.2–23.6) | 1.5 (0–9.1) | 1.4 (0–8.8) | 3.2 (0.1–19.1) | 1.3 (0–7.6) | 1.7 (0.1–9.3) |
| I | 4.8 (0.1–26.8) | 1.3 (0–8.6) | 1.2 (0–8.4) | 3.1 (0.1–17.9) | 0.9 (0–6.4) | 1.4 (0–9.4) |
| J | 1.7 (0–11.4) | 0.7 (0–6.3) | 1.1 (0–8.8) | 2.2 (0–16.3) | 1.0 (0–7.3) | 1.2 (0–9.8) |
| K | 1.6 (0–13) | 0.7 (0–5) | 1.1 (0–8.6) | 1.6 (0–12.1) | 0.6 (0–5.2) | 1.3 (0–9.7) |
| Total | 5.8 (3.1–9.6) | 2.3 (1.1–4.1) | 2.1 (0.9–4) | 5.7 (2.9–9.5) | 2.3 (1.1–4) | 2.3 (1–4.1) |

*Values represent % (95%CI) of students infected on-campus across 300 simulations for each school. The prorated and optimal allocations both correspond to total testing capacity every 14 days.
†Cumulative on-campus attack rates when each school has a different transmission rate.
‡Cumulative on-campus attack rates when all schools have the same transmission rate ($R_0$ = 1.0).

**Appendix Table 5.** Expected proportion of students infected on-campus over 10 weeks under different scenarios in a model for COVID-19 test allocation strategy to mitigate SARS-CoV-2 infections across school districts*

| | | Cumulative incidence rate, % (95% CI) | | |
|---|---|---|---|---|
| Quarantine strategy | Testing frequency, d | Low transmission | Moderate transmission | High transmission |
| Household + class† | None | 4.0 (0.8–9.1) | 9.3 (1.8–19.6) | 18.2 (4.8–35.7) |
| | 28 | 2.3 (0.6–5.1) | 4.2 (0.7–9.2) | 7.0 (2.1–14.6) |
| | 21 | 2.0 (0.3–4.8) | 3.7 (0.8–8.0) | 5.5 (1.3–12.1) |
| | 14 | 1.5 (0.2–3.3) | 2.9 (0.6–6.2) | 4.1 (1.0–8.3) |
| | 7 | 1.0 (0.1–2.5) | 1.5 (0.2–3.8) | 2.1 (0.3–4.8) |
| Household only‡ | None | 6.1 (1.1–15.1) | 19.4 (2.9–41.5) | 50.0 (21.1–80.8) |
| | 28 | 3.7 (0.6–9.2) | 10.5 (1.9–24.5) | 26.6 (1.6–52) |
| | 21 | 3.3 (0.5–8.7) | 8.8 (1.0–23.4) | 22.3 (3.6–49.4) |
| | 14 | 2.8 (0.4–7.0) | 6.3 (0.8–15.6) | 14.7 (1–35.2) |
| | 7 | 1.2 (0.1–3.2) | 2.1 (0.2–5.4) | 3.4 (0.4–8) |

*Each column represents a different in-school $R_0$. We show results for increasing testing frequencies, from no testing to weekly testing.
†Class and households quarantined when a person tests positive.
‡Only households quarantined when a person tests positive.

**Appendix Table 6.** Expected proportion of school days students miss or are in school while infected for a school with moderate transmission rate in a model for COVID-19 test allocation strategy to mitigate SARS-CoV-2 infections across school districts*

| | | Proportion of school days, % (95% CI) | | |
|---|---|---|---|---|
| Quarantine strategy | Testing frequency, d | At school infected | Missed school not infected | Missed school infected |
| Household + class† | None | 1.4 (0.3–2.8) | 5.3 (1.3–10.5) | 0.6 (0.1–1.3) |
| | 28 | 0.6 (0.1–1.2) | 6.5 (2.0–12.0) | 0.6 (0.1–1.2) |
| | 21 | 0.5 (0.2–1.0) | 6.8 (2.6–11.9) | 0.5 (0.2–1.0) |
| | 14 | 0.4 (0.1–0.7) | 7.3 (2.9–12.1) | 0.5 (0.2–0.9) |
| | 7 | 0.2 (0.1–0.3) | 7.4 (3.4–12.0) | 0.5 (0.2–0.8) |
| Household only‡ | None | 3.0 (0.5–6.5) | 0.6 (0.2–0.9) | 0.4 (0.1–0.7) |
| | 28 | 1.6 (0.4–3.5) | 0.7 (0.3–1.3) | 0.6 (0.2–1.2) |
| | 21 | 1.3 (0.3–2.8) | 0.8 (0.3–1.4) | 0.6 (0.2–1.2) |
| | 14 | 0.8 (0.2–1.8) | 0.8 (0.4–1.4) | 0.6 (0.2–1.3) |
| | 7 | 0.3 (0.1–0.5) | 0.6 (0.3–1.0) | 0.5 (0.2–1.0) |

*Each column represents a different type of day. We show results for increasing testing frequencies, from no testing to weekly testing.
†Class and households quarantined when a person tests positive.
‡Only households quarantined when a person tests positive.

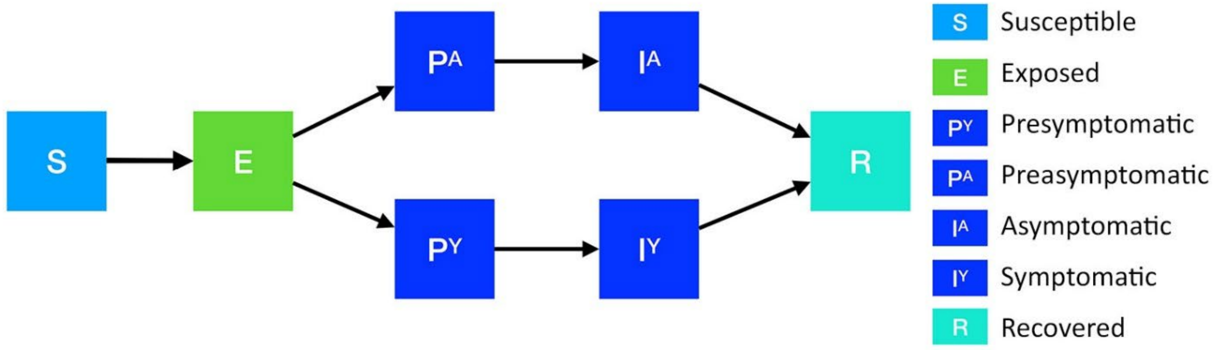


**Appendix Figure 1.** Schematic of the agent-based SEPIR model used for a COVID-19 test allocation strategy to mitigate SARS-CoV-2 infections across school districts. Upon infection, susceptible individuals (S) progress to exposed (E) and then to either pre-symptomatic infectious (PY) or pre-asymptomatic infectious (PA), from which they move to symptomatic infectious (IY) and asymptomatic infectious (IA), respectively. All cases eventually progress to a recovered class, where they remain protected from future infection (R). The proportion of individuals who become asymptomatic rather than symptomatic varies between children and adults. SEPIR, susceptible-exposed-presymptomatic-infectious-recovered.

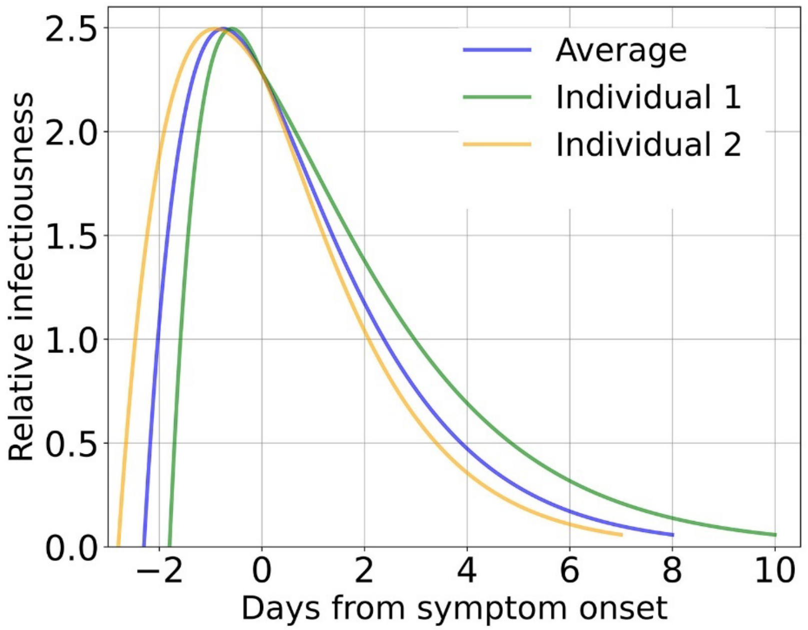


**Appendix Figure 2.** Relative infectiousness profile in a model for COVID-19 test allocation strategy to mitigate SARS-CoV-2 infections across school districts. The graph shows infectiousness through time of 3 infected persons relative to symptom onset. The average person (blue line) stays in the presymptomatic or preasymptomatic compartment for 2.3 days and the asymptomatic or symptomatic compartment for 8 days. Individual 1 spends 1.8 days the presymptomatic and 10 days in the infectious compartments. Individual 2 spends 2.8 in the presymptomatic and 7 days in the infectious compartments.

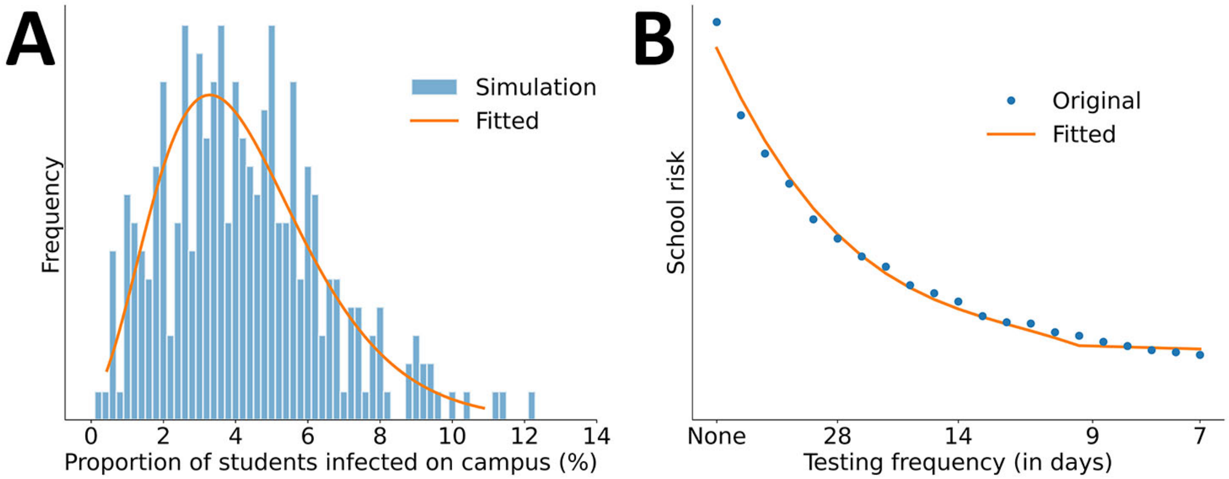


**Appendix Figure 3.** Processing results from the transmission model for COVID-19 test allocation strategy to mitigate SARS-CoV-2 infections across school districts. Processing results provide inputs to the optimization model. In both graphs the school used has an unmitigated $R_0$ of 1.5, daily community incidence of 35 cases per 100,000 population, and perfect COVID-19 tests are used. A) Shows frequency after fitting a gamma distribution to the results of the simulation model. The blue bars represent the number of students infected on a campus across 300 simulations. The orange line represents the best fitted nonnegative gamma distribution. Results correspond to a testing frequency of once every 4 weeks. B) School risk based on testing frequency. The graph shows fitting of a non-increasing curve to the risk of a school as a function of the proportion of students tested weekly. The blue dots represent the risk levels calculated by using the fitted gamma distributions. The orange line represents the best fitted curve used as input to the optimization model. The specific risk metric used is half the sum of the on-campus infection rate’s expected value and 90% conditional value-at-risk.

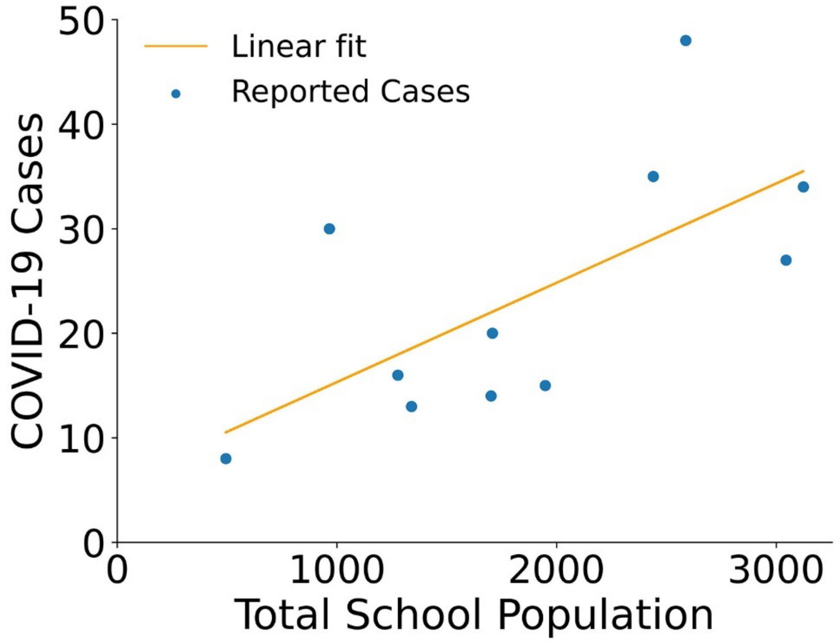


**Appendix Figure 4.** Reported COVID-19 cases in AISD high schools as a function of total school population used in a model for COVID-19 test allocation strategy to mitigate SARS-CoV-2 infections across school districts. COVID-19 cases were obtained from AISD's public dashboard (https://www.austinisd.org/dashboard) on March 8, 2021. School population includes total enrollment and staff. The orange line represents the best linear fit. AISD, Austin Independent School District

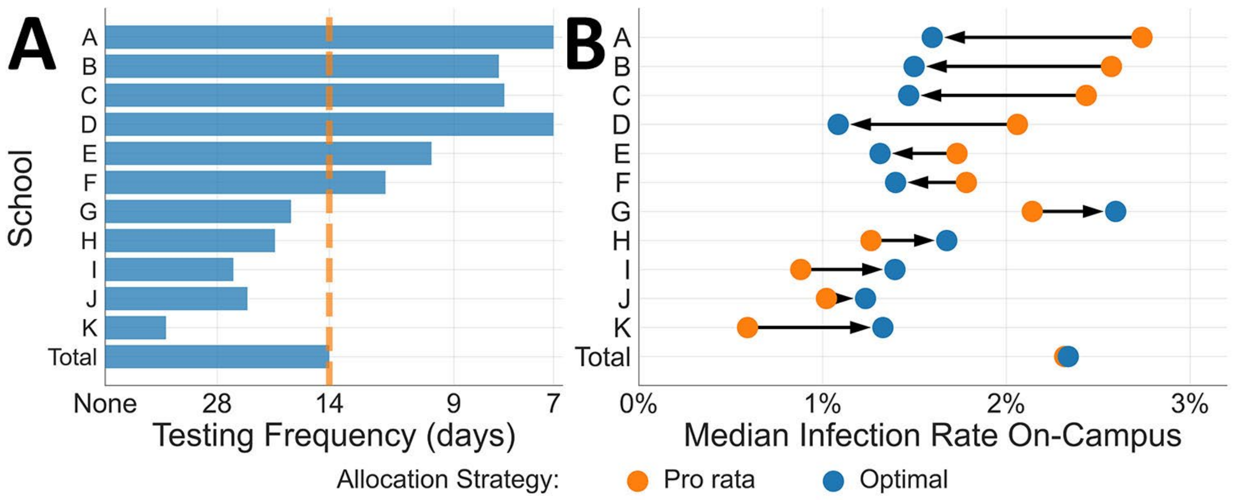


**Appendix Figure 5.** Testing frequency and median COVID-19 infection rate of 11 high schools in the Austin Independent School District used in a model for COVID-19 test allocation strategy to mitigate SARS-CoV-2 infections across school districts. A) Allocations for two testing strategies: pro rata, in which all schools test their students once per every 14 days (dashed orange line); optimized to minimize the maximum risk of any school, considering variation in community risk (blue bars). B) The median percent of students infected on-campus under the optimized strategy (blue) and pro rata strategy (orange), over a 10-week period. Arrows indicate increase or decrease in infection rate going from the pro rata to the optimal strategy. The model assumes all schools have the same transmission risk. Values are averages across 300 simulations. The model assumes that classrooms quarantine for 14 days after a positive test.

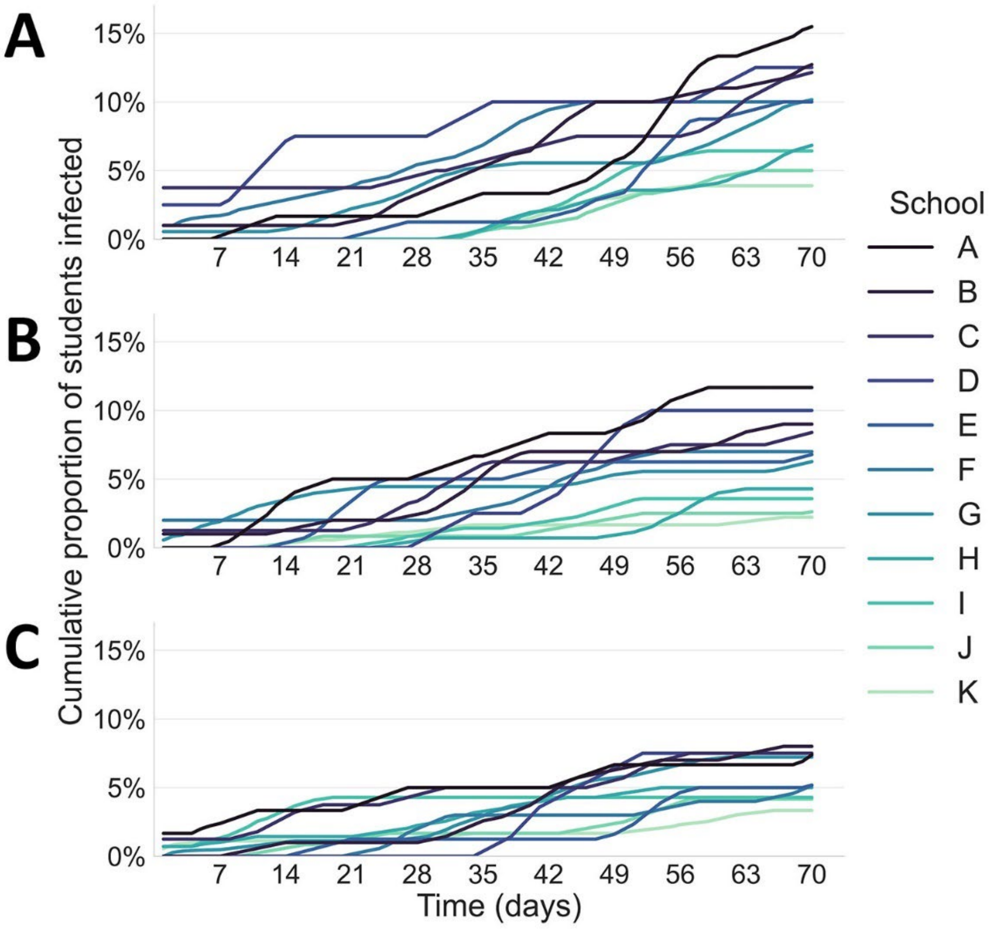


**Appendix Figure 6.** Cumulative proportion of students infected in a modeled COVID-19 test allocation strategy to mitigate SARS-CoV-2 infections across school districts. The graphs represent 3 testing scenarios: A) No testing; B) all schools test all students every 14 days; C) optimized allocation of tests based on schools having the same transmission risk, assuming a district-wide budget of one test per student every 14 days. Graphs show 11 high schools in the Austin Independent School District over a 10-week period. Schools are ordered from A–K based on community incidence in the school catchment area (from high to low). Graphs depict 7-day moving averages based on a single simulation for each scenario and school. To show representative projections, we selected the simulation that produced a cumulative attack rate closest to the median across all 300 simulations.

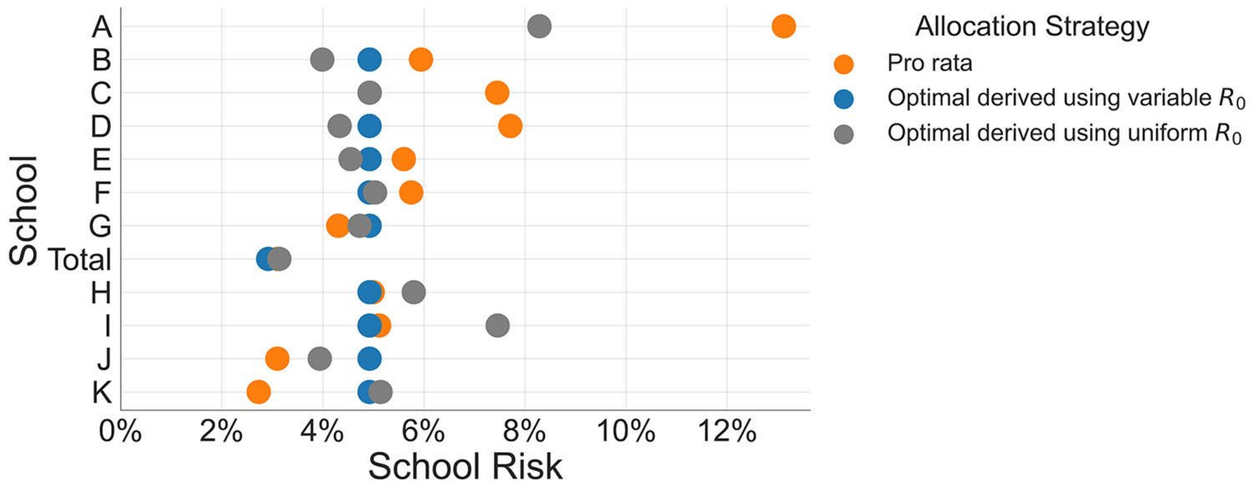


**Appendix Figure 7.** COVID-19 risk for schools used in a model for COVID-19 test allocation strategy to mitigate SARS-CoV-2 infections across school districts. We applied the model to 11 high schools in the Austin Independent School District. The graph shows the risk level of each school (A–K) under 3 possible testing allocations and a 14-day testing capacity when schools have different on-campus transmission rates. Risk was the objective value minimized in the optimization problem (*P*), defined as the average of the expected cumulative incidence (i.e., the mean across 300 simulations) and the projected tail risk. Blue dots correspond to the risk level under the optimal allocation obtained when we correctly assigned different on-campus transmission rates to each school. Gray dots correspond to the risk level obtained when the optimal allocation is obtained assuming all schools have the same on-campus reproduction number $R_0$ of 1.0. Orange dots correspond to the risk level under pro rata testing, in which all schools test their students once per every 14 days.

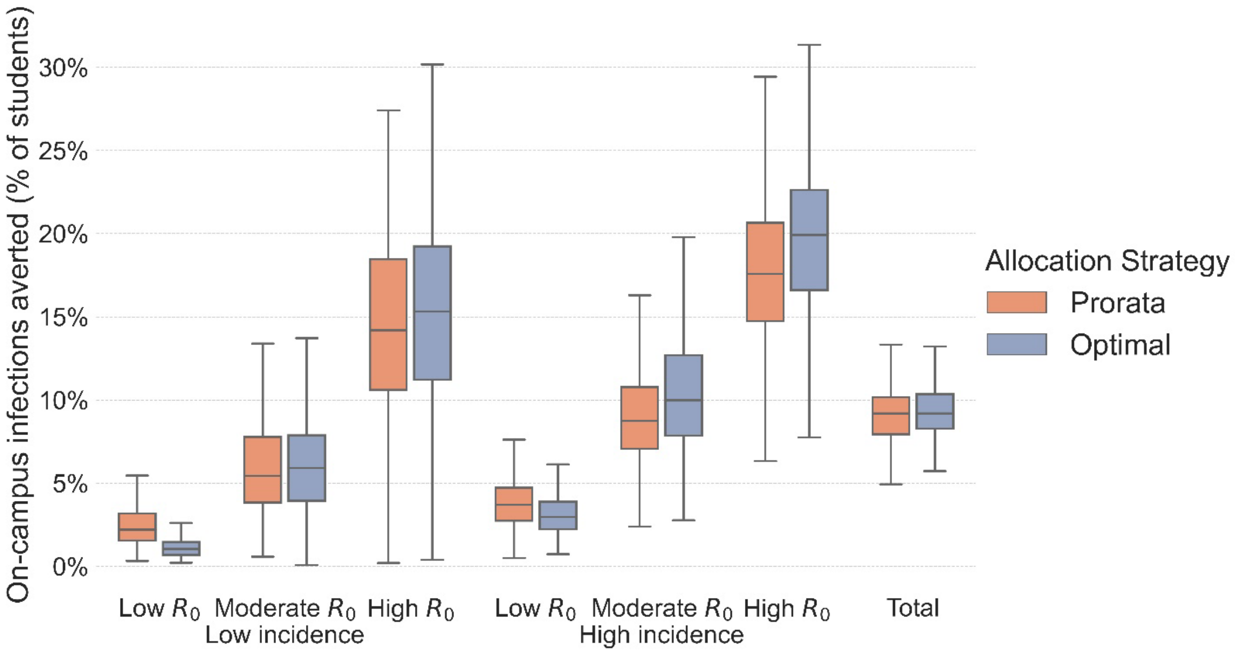


**Appendix Figure 8.** Proportion of on-campus infections averted in a model for COVID-19 test allocation strategy to mitigate SARS-CoV-2 infections across school districts. Averted on-campus infections are relative to no surveillance testing, for each of 6 different schools and the entire system over a 10-week period under 2 possible testing allocation strategies. In the modeled scenario, both allocation strategies have a budget to test students every 14 days. In the pro rata allocation, all schools test students every 14 days. When an individual tests positive, their entire classroom and household are quarantined for 14 days. In the optimal strategy, tests are allocated to minimize the maximum risk of any school, considering variation in community and in-school transmission risks. Boxes indicate interquartile range (IQR); horizontal bars inside boxes indicate median; whiskers indicate points that lie within 1.5 IQRs of the closest quartile.

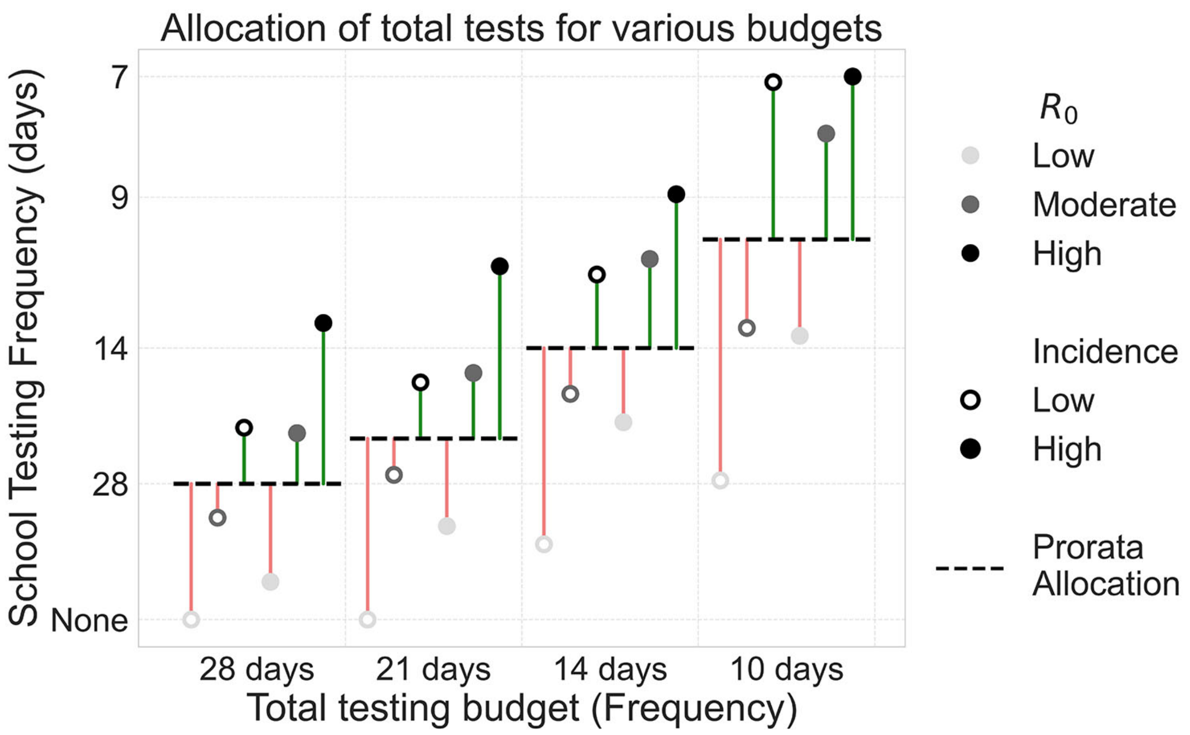


**Appendix Figure 9.** Optimal test allocations under different budgets in a model for COVID-19 test allocation strategy to mitigate SARS-CoV-2 infections across school districts. The horizontal dotted lines represent the prorated allocation for each budget. A green line indicates that a school receives more tests under our optimal allocation than under the prorated one, and a red line indicates a school receives fewer tests than the optimal allocation. Transmission risk has an $R_0$ value corresponding to 1.0 (low risk), 1.5 (moderate risk), 2.0 (high risk). Low community incidence is set at 35 new daily infections per 100,000 population; high community incidence is set at 70 new daily infections per 100,000 population. $R_0$, basic reproduction number.

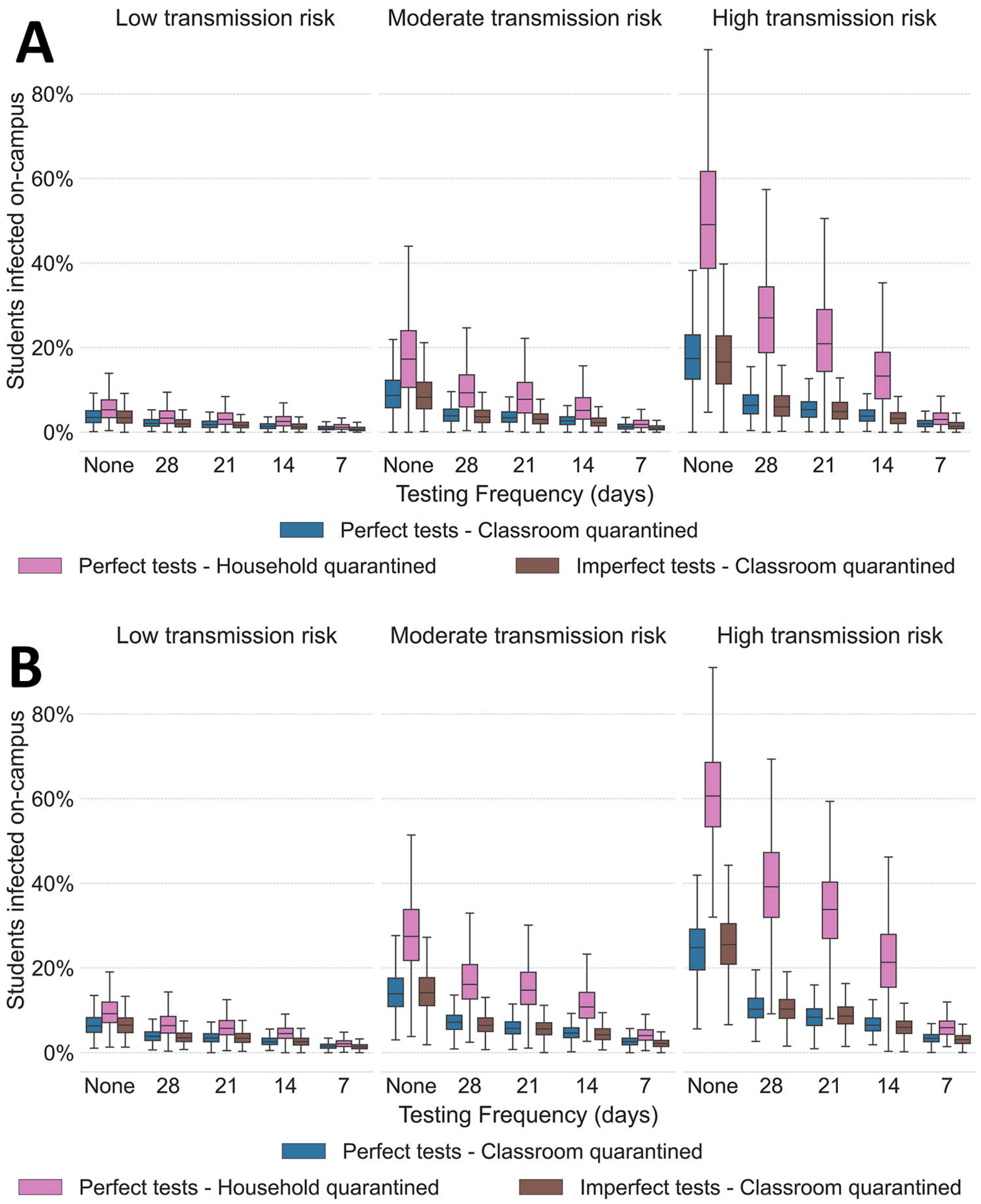


**Appendix Figure 10.** Projected effects of proactive SARS-CoV-2 testing in a model for COVID-19 test allocation strategy to mitigate SARS-CoV-2 infections across school districts. A) Low community incidence (35 new daily infections per 100,000 population). B) High community incidence (70 new daily infections per 100,000 population). We modeled fraction of students infected on campus depending on the frequency of proactive testing (none, or once per every 28, 21, 14, or 7 days) over a 10-week period in a school with 500 students. We modeled 3 scenarios (colors): entire classrooms are quarantined for 14 days after a positive test, by using either perfect or imperfect tests, or only households of persons testing

positive are quarantined for 14 days with perfect tests. Imperfect tests have a sensitivity of 95% for symptomatic individuals, 80% for asymptomatic (including pre-symptomatic) people, and we used a specificity of 99%, while perfect tests have 100% sensitivity and specificity. Transmission risk has an $R_0$ value corresponding to unmitigated basic reproduction numbers of 1.0 (low risk), 1.5 (moderate risk), 2.0 (high risk). The results are based on 300 stochastic simulations for each scenario. Boxes indicate interquartile range (IQR); horizontal bars inside boxes indicate median; whiskers indicate points that lie within 1.5 IQRs of the closest quartile. $R_0$, basic reproduction number.

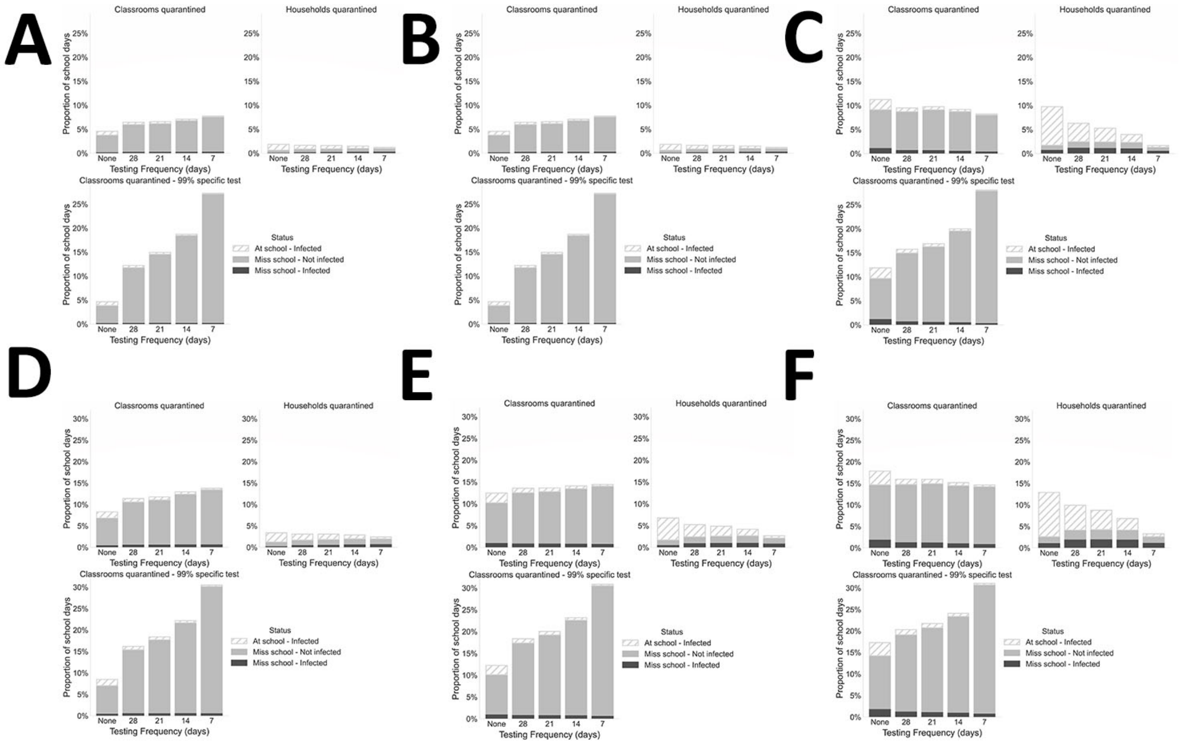


**Appendix Figure 11.** Effects of student testing on in-person attendance in a model for COVID-19 test allocation strategy to mitigate SARS-CoV-2 infections across school districts. A) Low community incidence (35 new daily infections per 100,000 population) and low transmission risk ($R_0$ = 1.0). B) Low community incidence and moderate transmission risk ($R_0$ = 1.5). C) Low community incidence and high transmission risk ($R_0$ = 2.0). D) High community incidence (70 new daily infections per 100,000 population) and low transmission risk ($R_0$ = 1.0). E) High community incidence and moderate transmission risk ($R_0$ = 1.5). F) High community incidence and high transmission risk ($R_0$ = 2.0). We modeled student attendance depending on the frequency of proactive testing (none, or once per every 28, 21, 14, or 7 days) over a 10-week period in schools with 500 students based on a total of 300 simulations for each scenario. Graphs show average number of school days missed due to isolation while infected, or while quarantined and disease-free, as well as average number of days in school while infected, depending on

the testing frequency. The top row of each figure corresponds to perfect tests where either entire classrooms or households only are quarantined after a positive test. The bottom rows correspond to tests with 99% specificity, 80% sensitivity for asymptomatic persons, and 95% sensitivity for symptomatic individuals, and entire classrooms quarantined after a positive test.

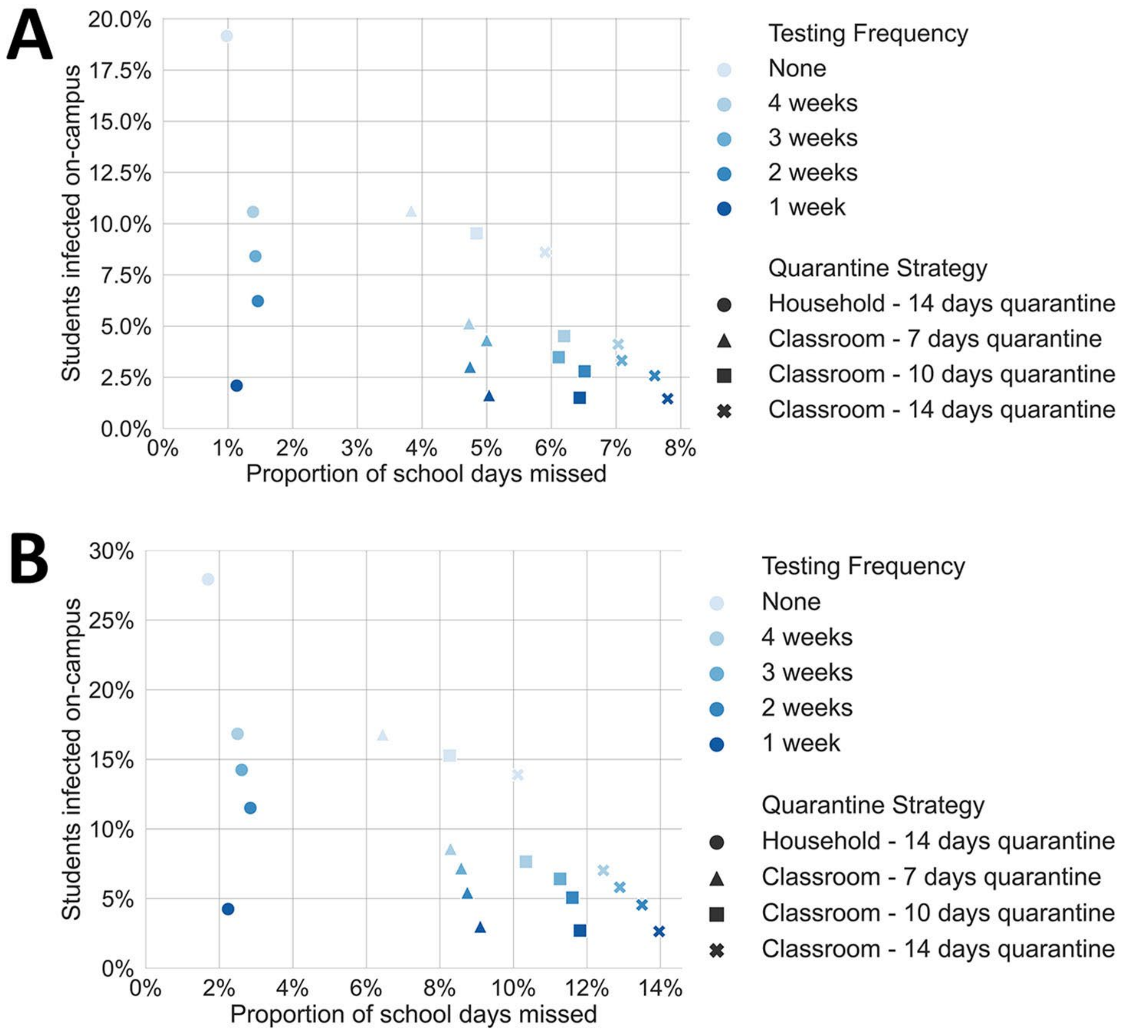


**Appendix Figure 12.** Tradeoff between infections prevented and school days missed for different quarantine strategies in a model for COVID-19 test allocation strategy to mitigate SARS-CoV-2 infections across school districts. A) Low community incidence (35 new daily infections per 100,000 population). B) High community incidence (70 new daily infections per 100,000 population). Each point represents the average proportion of students infected on-campus (vertical axis) and the average proportion of school days missed (horizontal axis) for 300 simulations over a 10-week horizon. Schools have an unmitigated $R_0$ of 1.5. $R_0$, basic reproductive number.

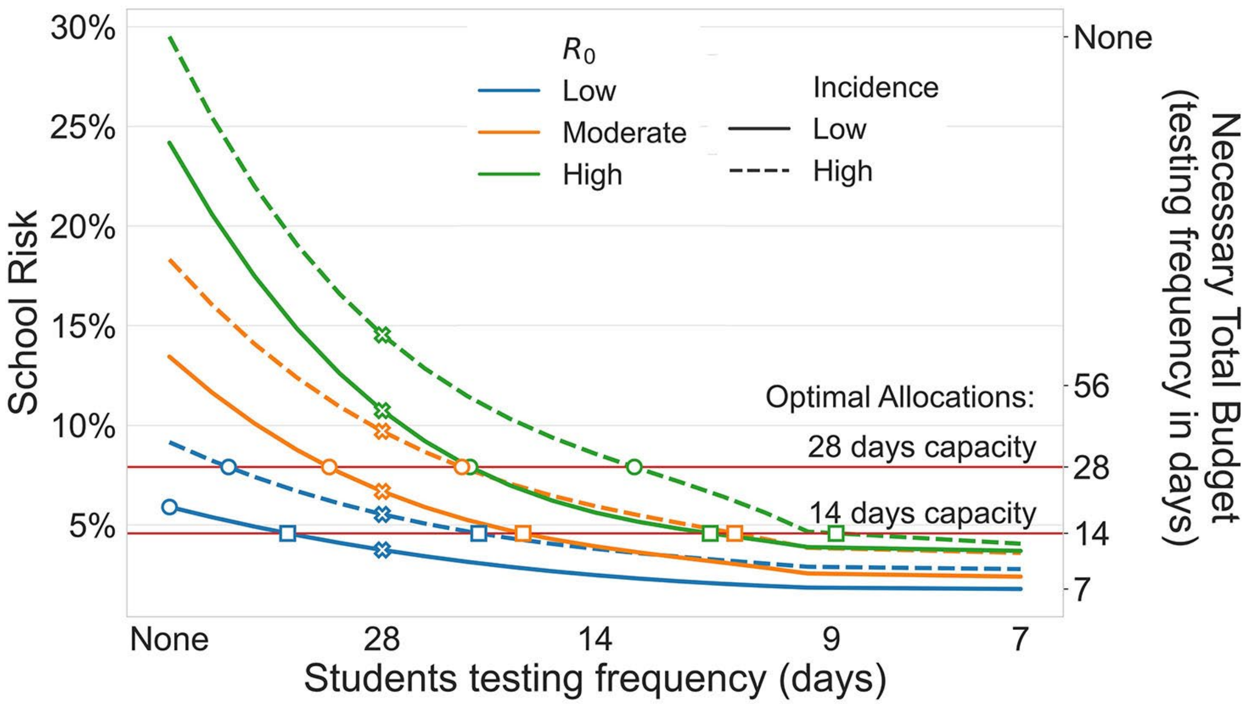


**Appendix Figure 13.** Individual school risks as testing frequency increases in a model for COVID-19 test allocation strategy to mitigate SARS-CoV-2 infections across school districts. The left axis indicates individual school risk for a given testing frequency. The right axis indicates the budget necessary to achieve a given risk level. The horizontal lines explicitly show the minimal risk attainable for system-wide testing capacities of 4 weeks and 2 weeks, with the circles and squares representing the schools' risk and testing level under the optimal allocations for these two budgets. The crosses represent the risk and allocation of each school under a prorated allocation of an every 4-weeks testing budget. Transmission risk has a value corresponding to unmitigated an $R_0$ of 1.0 (low risk), 1.5 (moderate risk), or 2.0 (high risk). Low community incidence corresponds to 35 new daily infections per 100,000 population and high community incidence corresponds to 70 new daily infections per 100,000 population. $R_0$, basic reproductive number.

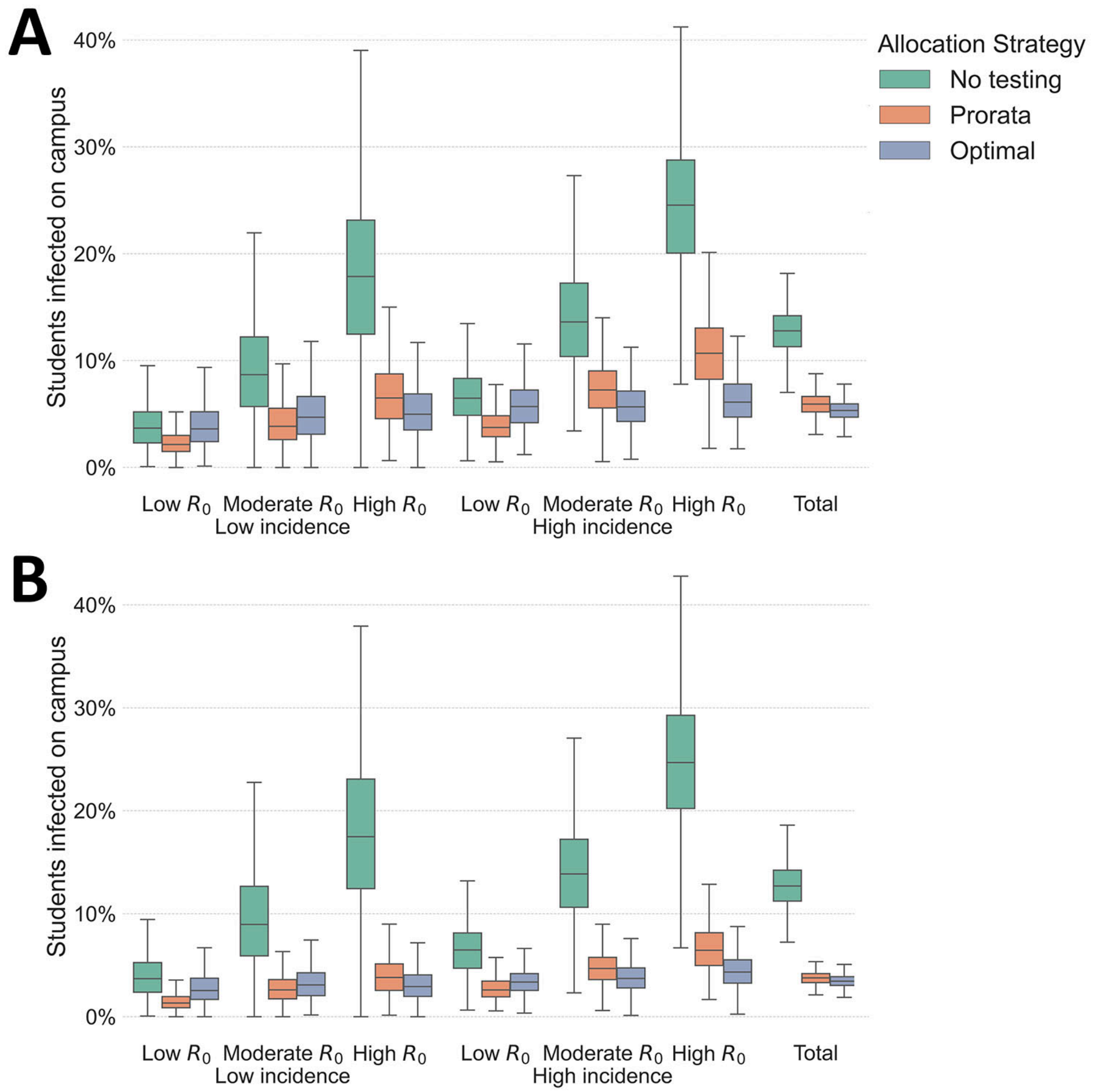


**Appendix Figure 14.** Distribution of the proportion of students infected on-campus in each school and in the entire school system in a model for COVID-19 test allocation strategy to mitigate SARS-CoV-2 infections across school districts. We modeled infections over a 10-week period under 3 possible testing strategies. The pro rata and optimal allocations use the same total testing budget for a testing frequency of every 4 weeks (A) or for a for a testing frequency of every 2 weeks (B). Boxes indicate interquartile range; horizontal bars inside boxes indicate median; whiskers indicate points that lie within 1.5 IQRs of the closest quartile. Transmission risk has a value corresponding to unmitigated an $R_0$ of 1.0 (low risk), 1.5 (moderate risk), or 2.0 (high risk). Low community incidence corresponds to 35 new daily infections per 100,000 population and high community incidence corresponds to 70 new daily infections per 100,000 population. $R_0$, basic reproductive number.

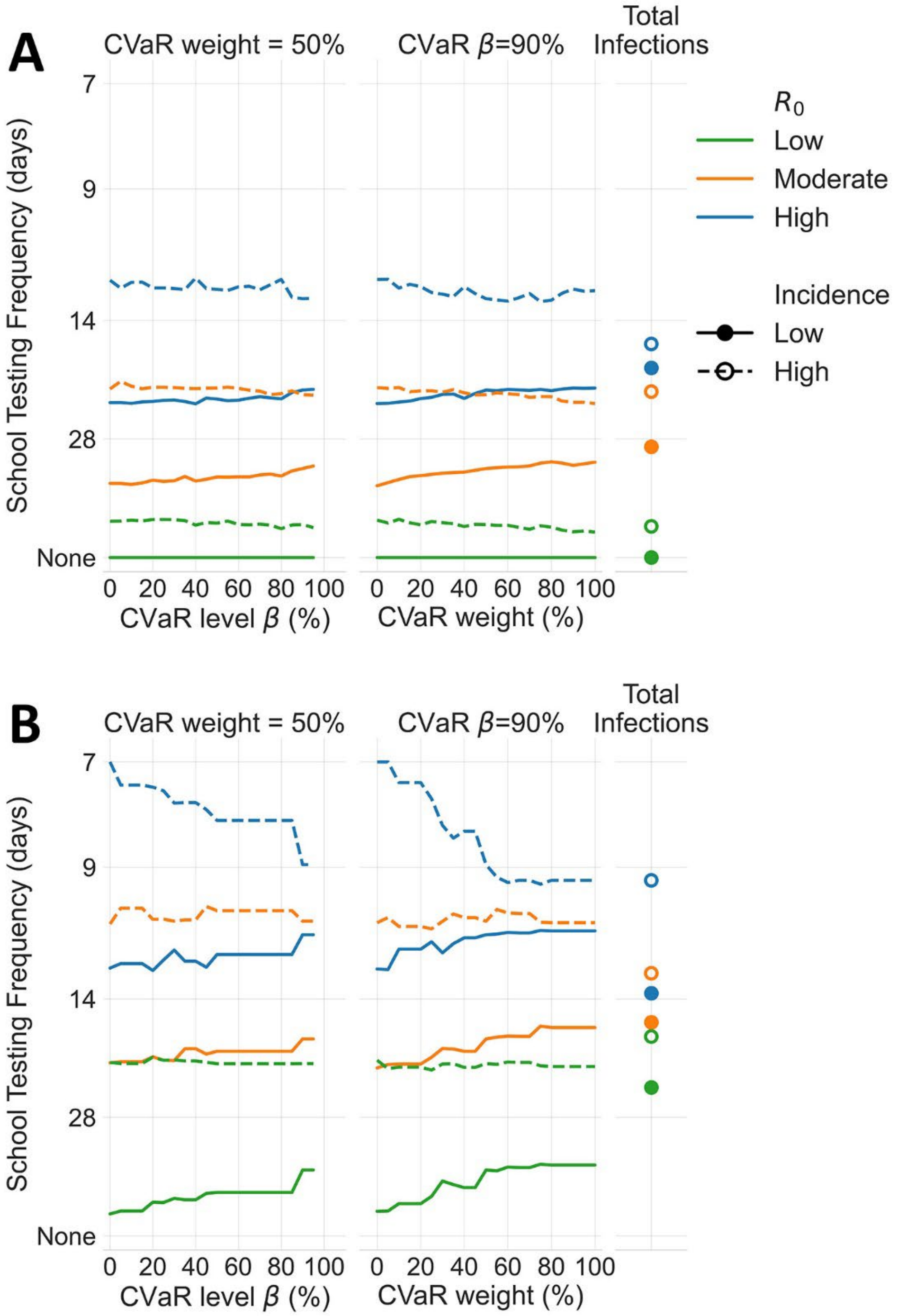


**Appendix Figure 15.** Optimal allocation to each school for different objective functions in a model for COVID-19 test allocation strategy to mitigate SARS-CoV-2 infections across school districts based on budgets for a testing frequency of 4 weeks (A), or for a testing frequency of 2 weeks (B). The left panels correspond to an objective function where the relevant quantile for CVaR increases from the 5% quantile to the 95% quantile, with the weight on the CVaR fixed at 50% (with the other 50% on the number of expected infections). The center panels correspond to an objective function where the weight of CVaR

term grows from 0% (expectation only) to 100% (CVaR only), with the CVaR level fixed at 90%. The right panels correspond to the optimal allocation obtained from minimizing the total number of on-campus infections across schools. Transmission risk has an $R_0$ value corresponding to unmitigated basic reproduction numbers of 1.0 (low risk), 1.5 (moderate risk), or 2.0 (high risk). CVaR, conditional value-at-risk; $R_0$, basic reproduction number.
